# 3D technologies for intangible cultural heritage preservation—literature review for selected databases

**DOI:** 10.1186/s40494-021-00633-x

**Published:** 2022-01-04

**Authors:** Maria Skublewska-Paszkowska, Marek Milosz, Pawel Powroznik, Edyta Lukasik

**Affiliations:** Department of Computer Science, Lublin University of Technology, Nadbystrzycka 38D, Lublin, Poland

**Keywords:** Intangible cultural heritage, 3D technologies, Literature analysis

## Abstract

Conservation of cultural heritage is nowadays a very important aspect of our lives. Thanks to such legacy we gain knowledge about our ancestors, methods of production and ways of their life. The rapid development of 3D technology allows for more and more faithful reflection of this area of life. The rich cultural heritage, both tangible and intangible, can be preserved for future generations due to the use of advanced 3d technologies. They provide the means of documenting, recovering and presenting items of cultural heritage. Not only buildings or monuments are taken into account. An important aspect of our culture is intangible cultural heritage (ICH), including acting, crafting or storytelling, passed down from generation to generation. Due to the rapid development of civilisation and the migration of people, this type of culture is often forgotten. That is why the preservation of ICH is an important element of today world. The main aim of this study, on the basis of the gathered papers, is to identify: (1) the general state of use of 3D digital technologies in ICH; (2) the topics and themes discussed; (3) the technologies used in the study; (4) locations of research centres conducting such studies; and (5) the types of research carried out. The methodology consists of the following main steps: defining study questions, searching query development, selection of publications in Scopus, Web of Knowledge and IEEE Xplore, finally the study execution and the analysis of the obtained results. The results show that for ICH the most often used technologies are: 3D visualisation, 3D modelling, Augmented Reality, Virtual Reality and motion capture systems.

## Introduction

The definition of cultural heritage (CH) has been changing together with the world developments in conservation [1, 2]. According to the United Nations Educational, Scientific and Cultural Organisation (UNESCO), heritage has a very wide definition that includes both tangible cultural heritage (TCH) and intangible cultural heritage (ICH) [2]. The former focuses on monuments, collections of objects, archaeological findings and others [3–10]. UNESCO’s 2003 Convention for the Safeguarding of ICH defined five areas that belong to it [11]:

- oral traditions and expressions (e. g. language, storytelling);
- performing arts (e.g. singing, dancing, theatre, feasting);
- social practices, rituals and festive events;
- knowledge and practices concerning nature and the universe;
- knowledge and skills used to produce traditional crafts.

It must be stated that this definition of ICH is not a final one. There are different variations used locally in some countries, such as traditional plays and games, culinary traditions, animal husbandry, pilgrimage and places of memory.

The deliberate act of keeping cultural heritage from the present for the future is known as preservation, and is currently used in historical museums, cultural centres, scientific research, education and others [12]. One of the possibility to develop this process is application various 3D technologies. They allow to access to the culture heritage elements that are difficult to reach in a real world [10]. Preservation concerns the following fields of study: documentation, protection, reconstruction, restoration, conservation, dissemination and spreading. Documentation is connected with storing various types of information. Protection is defined as actions against damage, destruction or other loss of CH. Reconstruction is a process of visualisation of CH objects for their better understanding. Restoration is a set of actions including the following tasks: integration and replacement of non-original elements, reconstruction, retouching and infilling. Conservation is about extending the life of cultural heritage while strengthening the transmission of its significant heritage messages and values [13]. Dissemination concerns representation and visualisation of TCH and ICH objects using modern technology [14]. Spreading is for accessing a possibly widest group of recipients in order to get them acquainted with cultural heritage.

Usually, tangible culture tends to last much longer than intangible culture. As a result of archaeological discoveries, some or all of the material things used in the past are preserved. A much more difficult situation concerns the intangible aspects of cultural life, which are passed down from generation to generation. The analysed materials show that human migration is one of the causes the forgetting and modification of this kind of culture [15–17]. Stories and experience are often forgotten or inaccurately communicated. Civilisation changes also result in the loss of indigenous culture. That is why the archiving of non-material culture for future generations is currently such an important initiative. ICH can be stored using a variety of analogue data, text, and two-dimensional (2D) technologies. Sometimes, however, it is not enough. In addition, over time analogue technologies are exposed to a natural loss of the quality of documented ICH. Digital technologies support ICH documentation processes and provide safer, more unchangeable capture and collection of ICH data. Moreover, a combination of various 3D technologies results in a better understanding of the topic and brings a new insights into the study [18].

There is a noticeable increase in the importance of 3D technology in science in various fields. The aim of the work was to perform a literature review on applied 3D digital technologies in the processing, presenting and protection of data related to intangible cultural heritage. The study was conducted on the basis of the three most popular databases (Scopus, Web of Knowledge and IEEE Xplore). The authors wanted to find out the answers whether these technologies are used in ICH area, what are the most popular topics, which countries are at the fore in such studies, and what types of research are being carried out. To the authors’ knowledge, there is no such comparison regarding quantitative studies, geographic, technological and product cross-section.

3D digital technologies are used in studies concerning CH aspects, including 3D scanning, 3D modelling, Virtual Reality (VR) and Augmented Reality (AR) are often used to perform virtual presentations of monuments and ancient artefacts. 3D, 4D, motion capture systems allow for recording the way of performing activities, their consolidation and presentation for subsequent generations. Often digital technologies, e.g. audiovisual ones are combined with 3D techniques for better presentation of the elements of the culture. It is possible to register national dances (their successive sequences), perform traditional craft, pass the storytelling, ancient events or the knowledge about building in the ancient times. Despite their immateriality, these technologies make it possible to preserve this type of culture.

## Study motivation

This study originated in 2015, arising from cooperation with many science and culture institutions from Europe and Central Asia in the area of digitalisation of cultural heritage. During scientific expeditions, researchers observed a relationship between TCH and ICH e.g. scanning objects and the method of their creation. Contacts related to the digitalisation of TCH resulted in the expansion of the author’s interest in ICH. One of the first steps towards expanding scientific interest is the analysis of the state of science and practice in the area of applying digital 3D technologies in ICH.

State of the art was aimed to identify:

- the general state of use of 3D digital technologies in ICH,
- topics and themes presented in the studies on the use of 3D digital technologies in ICH,
- technologies used in the studies on the use of 3D digital technologies in ICH,
- locations of research centres around the world that conduct studies using 3D digital technologies in ICH,
- types of studies results in the 3D digital technologies in ICH area.

## Methodology

**Fig. 1:**
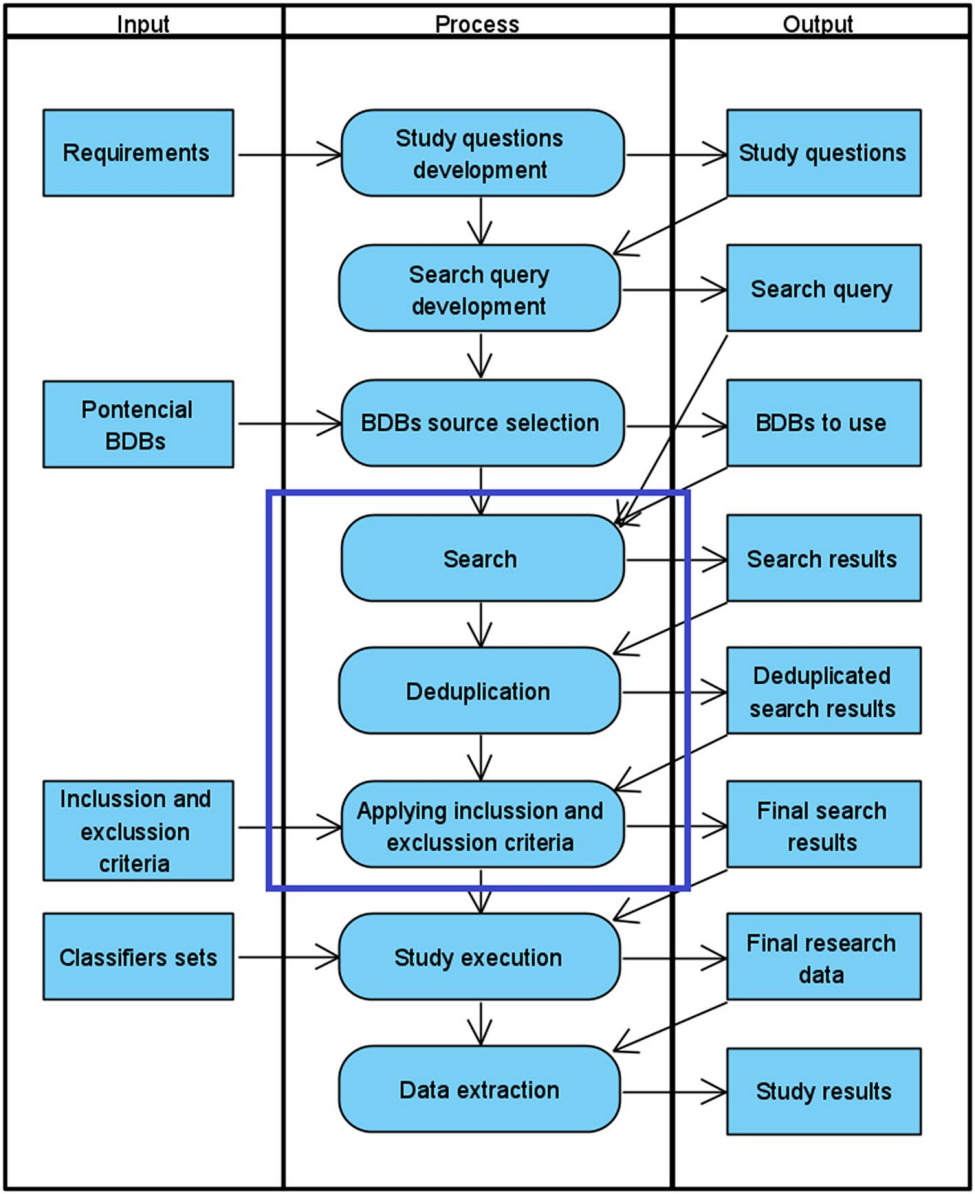
Study methodolody

The methodology designed and used in this study is presented in Fig. 1.

The methodology of a literature review of 3D Digital Technologies in Intangible Cultural Heritage consists of the following stages (the names of the processes come from Fig. 1):

1. Study questions (Qs) development. Based on the study objectives defined in section Study motivation, study questions are formulated, the answers to which will be sought during a literature review. They define the scope of the collected data and the analysis of the results.
2. Search query development—defining a query to bibliographic databases in order to search for publications containing the results of theoretical and applied studies in the area of 3D Digital Technologies in Intangible Cultural Heritage.
3. Bibliographic databases (BDBs) source selection. Selection of databases from available BDBS that will be used in a review.
4. Implementation of the search and selection of publications. This operation involves typical steps: publication search, deduplication of search results, and applying inclusion and exclusion criteria.
5. Study execution. This stage includes the analysis of the content of the publications selected in the previous stage. The analysis is carried out with the use of different sets of classifiers corresponding to the content of the Qs. The result is a database of publications assigned to different classifiers.
6. Data extraction – the analysis of the database created in stage 5.

### Study questions

Due to the lack of similar studies in the area of 3D Digital Technologies in Intangible Cultural Heritage, on the basis of the aims defined in chapter Study motivation, the following questions can be formulated:

**Q1.**: Is the use of 3D digital technologies in ICH developing now?
**Q2.**: What are the most important topics and themes presented in the studies on the use of 3D digital technologies in ICH?
**Q3.**: What technologies are used in studies on the 3D digital technologies in ICH area?
**Q4.**: What types of results in the 3D digital technologies in ICH area are presented?

### Search query

**Table 1:** Analysed words in the following databases

Words	Reasons
Cultural, folk	Formal synonym of culture
Heritage, legacy, inheritance	Formal synonym of heritage
Intangible, elusive, non-material	Formal synonym of intangible
3D, three-dimensional, three dimensional	Various methods of indicating 3D technology
History, past	General term to describe previous times

The query defined by the words based on Table 1 was as follows: TITLE-ABS-KEY: *(cultural OR folk) AND (intangible OR elusive OR non-material) AND (heritage OR legacy OR inheritance) AND (3D OR three-dimensional OR “three dimensional”) AND (history OR past)* and it was too detailed and returned only 16 studies. That is why “history” and “past” were excluded. The query *(cultural OR folk) AND (“intangible heritage” OR “elusive heritage” OR “non-material heritage”) AND (3D OR three-dimensional OR “three dimensional”)* returned also too low number of results. So the final query was defined as follows: *cultural AND (intangible OR non-material) AND heritage AND (3D OR three-dimensional OR “three dimensional”)*.

This query was adapted to the formats used in the selected bibliographic databases.

### Source bibliographic databases

The authors had access to the following bibliographic databases:

- InfonaNet;
- AccessEngineering;
- AIP / APS;
- EBSCOhost;
- EBSCO eBook;
- Emerald;
- IBUK Libra;
- EMIS Intelligence;
- IEEE Xplore;
- JCR;
- JSTOR;
- Medline;
- McGraw Hill eBook Library;
- Nature;
- MathSciNET;
- Science;
- Scopus;
- ScienceDirect;
- SpringerLink;
- Total Materia;
- Trans Tech Publications;
- Web of Knowledge;
- Wiley Online Library.

Three databases, the most prestigious in the field of technology, were selected for this purpose: Scopus, Web of Knowledge and IEEE Xplorer. These databases best fit the nature of the study in our area.

### Classifiers of scientific works used in the study

In stage 5 of our search (Fig. 1), the content of scientific publications selected in stage 4 (Fig. 1) was analysed in detail. Each of them was classified, i.e. the appropriate features were assigned from the sets of classifiers. The sets of classifiers were developed so that from the created database it was possible to answer the study questions through Data extraction (Fig. 1).

The following sets of publication content classifiers were used (* marked as extensible sets as the analysis of the content of the papers was initially defined, for which only the first few features were defined):

- **Set 1.** Cultural Heritage types: ICH and TCH & ICH.
- Set 2*. Keywords: Cultural Heritage, Intangible, 3D Modelling, Virtual Reality, Digitalisation.
- **Set 3.** Publication types: Conference Paper, Article, Book, Conference Review, Editorial, Letter.
- **Set 4.** Focus areas: Documentation, Protection, Reconstruction, Conservation, Dissemination, Spreading.
- Set 5*. Study fields: initial was empty.
- Set 6*. 3D technologies: 3D Modelling, Motion Capture, Virtual Reality, Augmented Reality, 3D Scanning, Photogrammetry.
- Set 7*. Aims of studies: Promoting Heritage, Recognition, Visual Reconstruction, Education.
- **Set 8.** Study results: Presentation, Virtual Exposition, Data Set, Web Application, AR Application, Museum, Mobile Application, Game.

## Detailed Literature Study

### 3D technologies in ICH

The development of 3D technology has made it possible to preserve ICH in various fields. A great number of studies concerns craft, dance, storytelling, game-based education and various types of application for educational and documentary purposes. It is stated that sport and settlement are also a widely described issues.

#### Craftsmanship studies

**Fig. 2:**
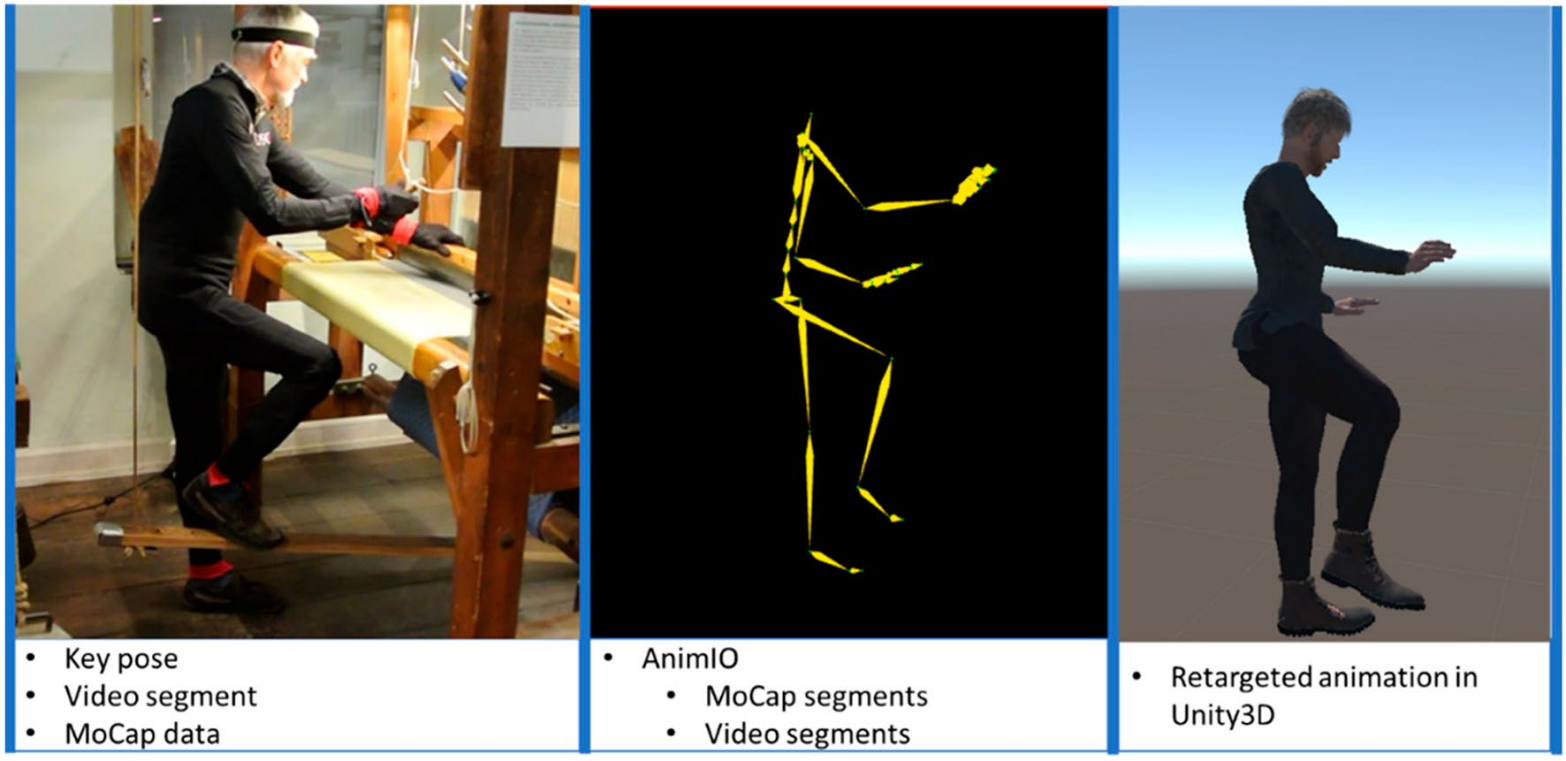
Steps taken to create a virtual representation of the craftsman at work [23]

Craftsmanship studies concern various types of traditional know-how of movements, human activities and manufacturing process. They involve tuna fishing [19], manufacturing authentic fabric in Transylvanian villages [20], Dongyang bamboo [21], traditional joinery technique [22], visualisation of the craftsman at work [23–25]—an example is presented in Fig. 2, artistic handicrafts in Lucchesia (Tuscany, Italy) [26], porcelain making crafts of Jingdezhen [27], traditional Chinese Gold Lacquer Wood Carving [28], sword motions—Japanese kenjutsu [29], traditional Tujia brocade skill [30] and the art of Franco Purini and Francesco Cellini [31]. Technologies used in the individual articles are presented in Table 2.

**Table 2:** Technologies used in craftsmanship studies

Papers	Technologies used
Repola et al. [19]	GIS-BIM System, 3D visualisation, 3D modelling
Cristin et al. [20]	3D visualisation, AR, 3D modelling
Wu et al. [21]	VR, AR, CAD, Unity 3D, motion capture
Rossau et al. [22]	VR, 3D modelling
Partarakis et al. [23]	Motion capture, VR, 3D modelling
Jeong et al. [24]	Motion capture, 3D modelling, AR
Koutsabasis et al. [25]	Motion capture, 3D visualisation, 3D modelling
Carrozzino et al. [26]	VR, 3D visualisation, 3D modelling
Wang et al. [27]	3D modelling, VR
Lou et al. [28]	3D printing
Aoki et al. [29]	Motion capture, VR
Zhao et al. [30]	Motion capture, 3D visualisation, 3D modelling
Farroni et al. [31]	3D visualisation, 3D modelling

Digital technologies allow to document, store and protect traditional crafting. 3D recording enables capturing movements. For this purpose various acquisition systems are used, such as Kinect or Leap motion. Collected data is presented in the form of 3D models, which are later visualised in many environments.

#### Art studies

**Table 3:** Technologies used in art studies

Papers	Technologies used
Rallis et al. [32]	Motion capture, 3D modelling, 3D photogrammetry
Voulodimos et al. [33]	Motion capture, 3D modelling
Voulodimos et al. [34]	Motion capture, 3D modelling, 3D visualisation
Douka et al. [35]	Motion capture, 3D modelling, 3D visualisation
Ziagkas et al. [36]	Motion capture, 3D modelling
Ziagkas et al. [37]	Motion capture, 3D modelling
Rallis et al. [38]	Motion capture, 3D modelling, VR
Douka et al. [39]	Motion capture, 3D modelling, 3D visualisation
Rallis et al. [40]	Motion capture
Rallis et al. [41]	Motion capture, 3D modelling
Rallis et al. [42]	Motion capture, 3D modelling
Lim et al. [43]	Motion capture, 3D modelling
Xiang et al. [44]	Motion capture, 3D modelling, Motion Builder
Ribeiro et al. [45]	Motion capture, 3D modelling, VR
Tongpaeng et al. [46]	Motion capture, 3D modelling, VR
Tongpaeng et al. [47]	3D modelling
Doulamis et al. [48]	Motion capture, 3D modelling, VR
Tongpaeng et al. [49]	3D modelling, 3D visualisation
Tongpaeng et al. [50]	3D modelling, 3D visualisation
Fu et al. [51]	Motion capture
Hajdin et al. [52]	Motion capture, 3D modelling, 3D visualisation
Stavrakis et al. [53]	Motion capture, VR
Rallis et al. [32]	Motion capture, 3D modelling
Hisatom et al. [54]	Motion capture, 3D modelling, 3D, photogrammetry
JI et al. [55]	3D modelling, 3D scanning, 3D visualisation

**Fig. 3:**
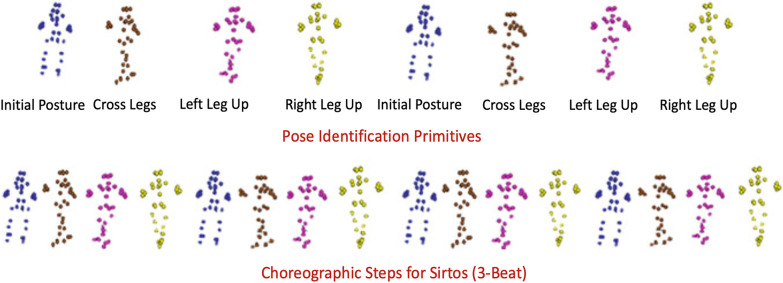
Visualisation of points from the motion capture tool used in archiving traditional Greek dances [32]

The subject of dance is widely discussed in articles on non-material culture. Its exemplary processing using motion capture tools is shown in Fig. 3.

Motion capture (both optical and based on accelerometers) and photogrammetry technology are often used for movement acquisition for various aspects of cultural, technical, academic, choreographic, spatial, commercial and educational purposes. The studies include traditional folk dances in Greece [32–42], Korea [43], China [44, 51], Portugal [45], South Asia [46], Thailand [47–50], Slovakia [52] and Cyprus [53] .

Research concerts modelling dance sequence trajectories, which allows to digitalise and analyse the recorded movements. The results are often used for 3D modelling (e. g. Motion Builder) and in Virtual Reality technologies. Many research works on ICH present the process of implementing a tool for translating dance notation into 3D animation, focusing on the whole body, hand or only finger movements. Technologies used in dance studies are presented in Table 3.

Based on obtained 3D data, sophisticated methods like machine learning, long short-term memory network or Bayesian Optimized Bi-directional Long Short-Term Memory are used for classification and comparison of various types of dances [32].

A special digital data acquisition system has been developed that can preserve Japanese traditional dramas in the form of dynamic 3D models [54]. The traditional costumes of Japanese traditional dramatic actors consist of a long-sleeved costume and a fan. The proposed method of obtaining 3D models from the motion capture system allows the reconstruction of thin and long elements.

ICH is also presented in [55] in the form of the virtual Art Gallery of Shanghai Style Lacquerware. 3D laser scanning technology, 3D modelling technology and 3DMAX were applied.

#### Storytelling studies

**Table 4:** Technologies used in storytelling studies

Papers	Technologies used
Selmanovic et al. [56]	VR, AR
Rizvic et al. [57]	VR, 3D modelling
Shih et al. [58]	3D modelling, 3D scanning, 3D visualisation
Yang et al. [61]	AR, 3D modelling
Thomopoulos [59]	3D modelling, 3D visualisation
Selmanovic et al. [60]	VR, 3D modelling, 3D visualisation
Yang [61]	3D modelling

Interactive digital storytelling is used to draw special attention to the historic items, places (e. g. Old Bridge in Mostar) [56, 57], events (e. g. the Chinese lantern festival) [58], myths [59] or arts [61]. For this purpose methods like VR, AR, 3D modelling or 3D visualisation are used (Table 4). Additional techniques, applied together with the above mentioned, are audiovisual technologies, machine learning and natural language processing. This kind of combinations enabled users to visualise myths, artworks and their connections in comprehensible ways.

#### Games-based learning studies

**Table 5:** Technologies used in games-based learning studies

Papers	Technologies used
Rallis et al. [40]	Motion capture
Cosovic et al. [62]	VR
Yilmaz et al. [63]	3D modelling, 3D visualisation
Dagnino et al. [64]	Motion capture
Kennedy [65]	3D visualisation
Bonenberger et al. [66]	VR, 3D GIS
Haddad et al. [67]	VR
Anastasovitis et al. [68]	VR, 3D modelling, 3D animation
Partarakis et al. [69]	VR, 3D modelling
Brusaporci [70]	3D modelling, 3D visualisation

Game-based learning is more and more often used for the virtual museums in order to raise culture awareness and motivate the public to visit cultural institutions [62]. Traditional skills and habits related to culture are documented by way of oral traditions and expressions. Game-based learning is also applied to dance choreography and posture analysis [40]. It is aimed at supporting the learning for promoting the protection of intangible culture (e. g. singing in Sardinia, St Andrews Cathedral) [65], explaining the cultural heritage to children [67, 69], explaining ancient technologies [68] or showing the connection between ICH and spatial geography [66]. Game-based learning is used for promoting singing, such as Human Beat Box [63] or Canto a Tenore [64]. In game-based learning studies various technologies are applied, like 3D modelling together with computer simulations, VR or 3D Geographic Information System (3D GIS) (Table 5). They allow to explain and explore how educational and multimedia heritage enables users to understand and appreciate cultural heritage [70].

#### Software studies

Software studies of ICH concerns many forms of research: music [71, 72], folk culture [73, 74], dance, singing, theatre, art [75, 76] and others [77]. The digitalisation allows an advanced analysis of the inscriptions and obtaining a complete and precise 3D models of the items. It is often further used to produce an interactive application. In software studies the following technologies are often used: 3D modelling (e.g. 3D MAX), 3D visualisation, 3D environment (e.g. Unity 3D), VR, motion capture systems and Reflectance Transformation Imaging (RTI) (Table 6). For the purpose of digital system studies a platform has been created, which is a new ground in education and knowledge transfer combining conventional learning procedures and sensorimotor learning through an interactive 3D environment (i-Treasure).

**Table 6:** Technologies used in software studies

Papers	Technologies used
Gaugne et al. [71]	3D modelling, 3D photogrammetry, RTI
Hu et al. [72]	3D modelling, 3D visualisation
Huang et al. [73]	AR, VR, motion caplture, Unity 3D
Yu et al. [74]	3D modelling, Unity 3D
Dimitropoulos et al. [75]	Motion capture
Dagnino [76]	3D visualisation, 3D modelling, AR
Cai et al. [77]	AR, 3D visualisation

#### Sport

**Fig. 4:**
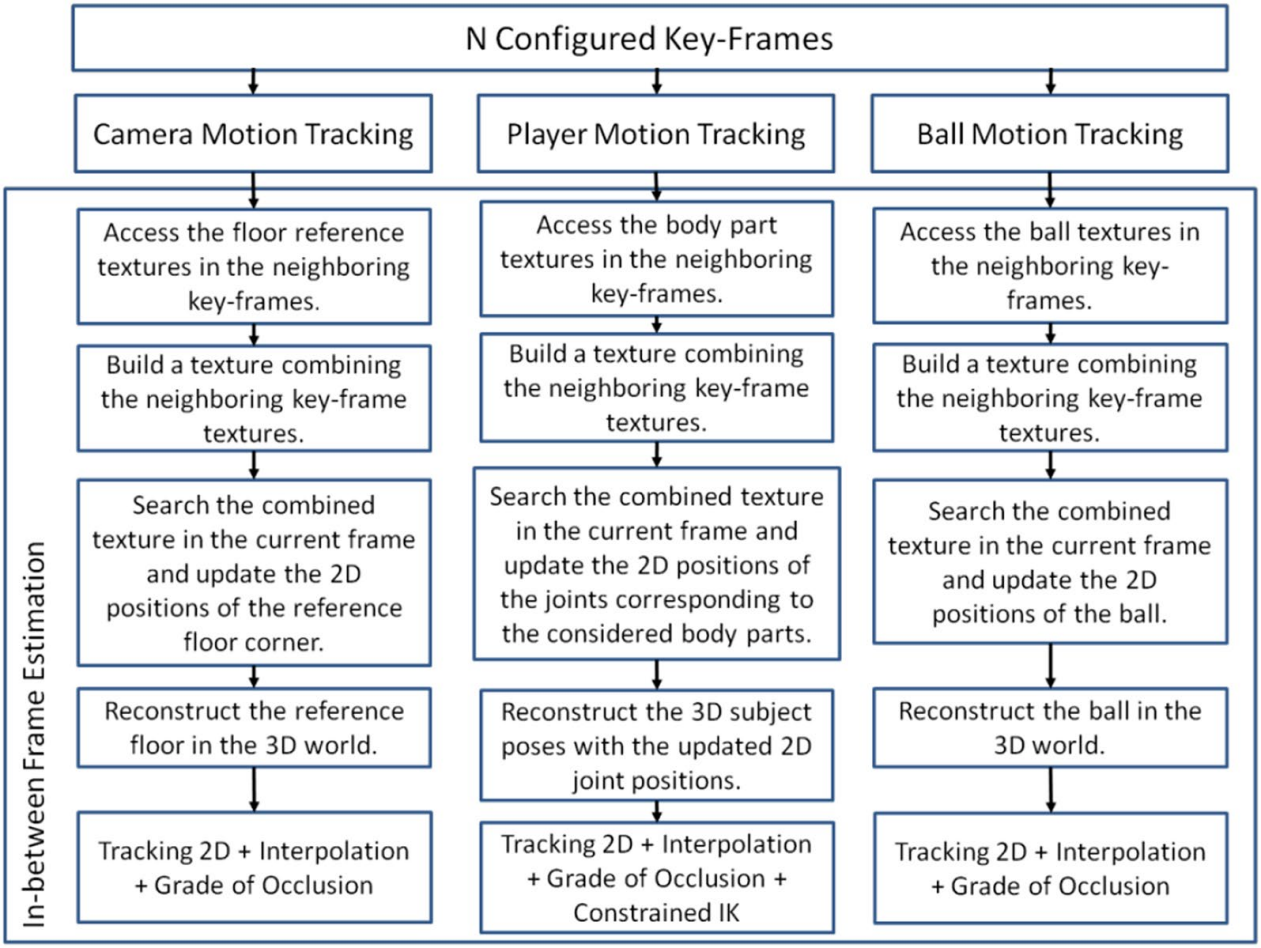
An example of In-between frame estimation method [78]

Sports are a key part of cultural identity and an important form of ICH. An additional challenge in processing of sports data is the need to capture the context of the development of athletes’ movement (together with their surroundings, including other objects, e.g. a ball) [78–80]. In many studies (Table 7) the use of motion capture technology in conjunction with other 3D techniques (e.g. Zbrush software, Autodesk, MPEG-4) allows not only to reproduce the behaviour of athletes, but also to visualise their appearance or outfit. One of the possible ways to capture the relationship between objects is to use the in-between frame estimation method presented in Fig. 4.

**Table 7:** Technologies used in sport studies

Papers	Technologies used
Goenetxea et al. [78]	Motion capture, 3D visualisation
Arevalo et al. [79]	Motion capture, 3D visualisation, 3D modelling
Linaza et al. [80]	Motion capture

#### Settlement studies

The aim of settlement studies is to aid the understanding and interpretation of ancient principles relating to sensitive and appropriate interaction of the built form and its associated landscape (e.g. First Nations) [81]. The principles have at their root the harmony of human inhabitation in the landscape they are surrounded by. A study of livelihood concerning the way of life based on environmental factors should also be taken into consideration [82–84]. To fulfil these aims the following technologies are usually used: 3D GIS, AR, 3D modelling and VR (Table 8).

**Table 8:** Technologies used in settlement studies

Papers	Technologies used
Tang et al. [81]	3D GIS
Jia et al. [82]	3D modelling
Yang et al. [83]	3D GIS, AR
Xu et al. [84]	VR

#### Other studies

**Table 9:** Technologies used in settlement studies

Papers	Technologies used
Yinfang et al. [85]	3D modelling
Yang et al. [86]	AR, 3D modelling
Shi et al. [87]	VR, 3D animation
Zhang et al. [88]	VR, 3D GIS, 3D modelling
Dai et al. [89]	VR, 3D modelling
Parrinello et al. [90]	VR, 3D modelling
Jung et al. [91]	Capture system
Li et al. [92]	3D modelling, 3D visualisation
Lassandro et al. [93]	3D modelling, 3D photogrammetry, 3D visualisation
Griffo et al. [94]	3D photogrammetry, 3D modelling, RTI
Gao et al. [95]	3D modelling, 3D visualisation, 3D printing
Manfren et al. [96]	3D auralization, 3D modelling
Alvarez-Morales et al. [97]	3D auralization
Xu et al. [98]	AR, 3D modelling, 3D visualisation

Despite the separation of previous research, there are articles that have been grouped as other studies. They concern a wide range of aspects of ICH, such as: explore the artistic style [85], modelling design [86], preservation of culture [87–89], languages, signs and symbols [90], conservation of traditional painting methods [91], reconstruction of ancient items (e.g. flame lighting systems) [92–95] or acoustic properties of the buildings (e.g. theatre in Bologna, catholic cathedrals in Spain) [96–98]. These areas involve such technologies as: AR, 3D modelling, 3D photogrammetry, VR, 3D printing, RTI and 3D auralisation (Table 9). Together with the methods mentioned above, the following techniques are used: laser scanner, structure from motion, infrared radiation or infrared thermography.

### Mixed culture heritage

A lot of studies focus on both material and non-material cultural aspects. They are closely related, complement and interpenetrate each other (Table 10).

#### Architecture studies

**Table 10:** Technologies used in architecture studies for mixed culture heritage

Papers	Technologies used
Wang et al. [99]	3D modelling, VR, 3D visualization
Banfi et al. [100]	3D photogrammetry, 3D modelling, VR
Themistocleous et al. [101]	3D visualization
Xue et al. [102]	3D modelling, 3D visualization
Sanchez-Aparicio et al. [103]	3D modelling, 3D visualization
Thomas et al. [104]	3D modelling, 3D visualization
Cao et al. [105]	3D modelling, 3D visualization, VR
Mallik et al. [106]	Motion capture, 3D visualization
Mallik et al. [107]	Motion capture, 3D visualization
Chroni et al. [108]	3D GIS, VR, 3D modelling
Tan et al. [109]	3D auralization
Balletti et al. [110]	3D modelling, 3D printing, 3D photogrammetry
Kitsikidis et al. [111]	Motion capture, 3D visualization, 3D modelling
Liu et al. [112]	3D modelling
Rodriguez-Gonzalvez et al. [113]	LIDaR

**Fig. 5:**
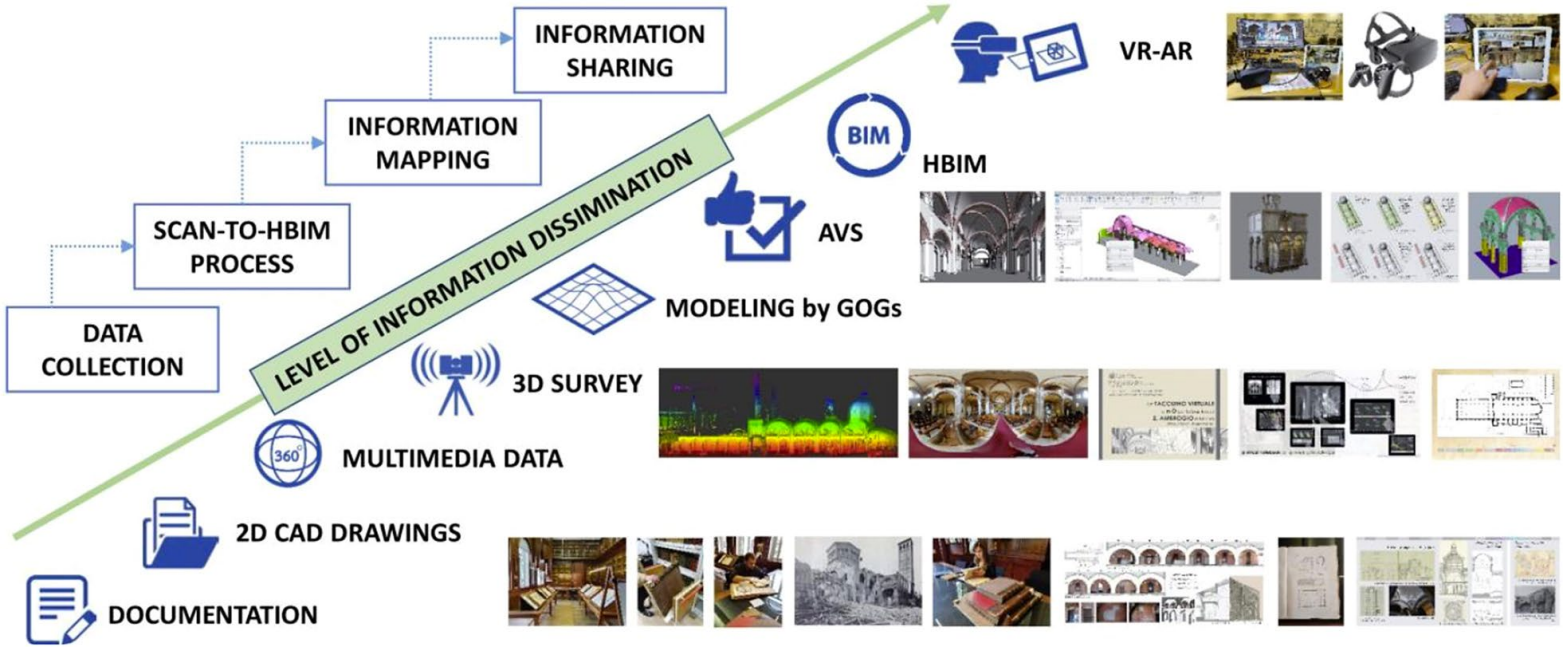
The research method applied to the XR project of Basilica of Sant’ Ambrogio [100]

Many papers concern both TCH and ICH. The arts integrated with historical analysis and iconographic sources are a perfect source of knowledge. The studies include historical architecture (e.g. Sant Marco Squere, Yuanming Yuan imperial Garden of the Qing Dynasty, Basilica of Sant’Ambrogio in Milan, churches and temples, Running Zhuma in Pizhou, Medieval Wall of Ávilath and others) [99–110]. They are extended by additional elements of culture such as: storytelling, the way of building monuments, headwear, clothing, music, soundscapes, spoken language, legends or interviews with inhabitants [111, 112]. Typically, in studies focusing on TCH and ICH, the following technologies are used: 3D modelling, VR, 3D visualisation, 3D auralisation, 3D GIS, motion capture, 3D photogrammetry or Light Detection and Ranging (LIDaR) (for large objects and sites) [113]. These methods are supported by Building Information Modelling (BIM), eXtended Reality (XR) and animation software (Autodesk, Maya 3D, Blender) in order to create interdisciplinary research. The methodology for creating AR using a big scene consisting of buildings, characters, tools and object is presented in Fig. 5.

#### Settlement studies

The settlement studies for a mixed type of culture concern historical sites (e.g. Zandieh Complex and Hafezieh Tomb) [114] as well as detection of the new archaeological places, digital repository about movable, stationary and immaterial heritage (e.g. the Danube region) [115], migrations (e.g. Paths of Roman Routes—Via Traiana in Italy, Via Egnatia in Albania, and Via Flavia in Albania and Montenegro) [116], memories and legends (e.g. the Ogiek people) [117] and reconstruction of behaviour of ancient people [118, 119]. They are often extended with the use of 3D objects, films, texts, photos and descriptions for better culture understanding. For the purpose of the study the following technologies are commonly used: 3D modelling, 3D GIS, 3D visualisation, VR, AR, 3D photogrammetry or LIDaR [120] (Table 11). They enables the interaction of visitors with a variety of informational content.

**Table 11:** Technologies used in settlement studies for mixed culture heritage

Papers	Technologies used
Chizfahm et al. [114]	3D modelling
Zakrajsek et al. [115]	3D GIS
de Fino et al. [116]	3D GIS, 3D visualization, VR, AR
Rambaldi et al. [117]	3D modelling
Cheng et al. [118]	VR, 3D modelling, 3D visualization
Yang et al. [119]	VR, 3D photogrammetry, 3D modelling
Coren et al. [120]	LIDaR

#### Museum studies

Another group of study is related to museum collections which are presented in a form of virtual exhibitions [121]. Artworks are characterised by intangible and historical values, such as heterogeneous documentary heritage that enlightened the need of creating new narratives while avoiding the descriptive and analytical ones [122]. For the purpose of the museum study the following technologies are commonly used: 3D modelling, VR, 3D photogrammetry and motion capture (Table 12). The created applications (mobile or web) are often based on specific frameworks (e.g. 3D Heritage Online Presenter). The paper’s aim is to improve the users’ view during their visits in the virtual museum [123].

**Table 12:** Technologies used in museum studies for mixed culture heritage

Papers	Technologies used
Wang et al. [121]	3D modelling, VR, 3D photogrammetry
Lo Turco et al. [122]	3D modelling, 3D photogrammetry, 3D visualization
LiI et al. [123]	Motion capture, 3D visualization, VR

#### Software studies

Many papers related to digital system studies are based on creating a platform consisting of websites, available from a smartphone, where sightseers can read the narrative or download various materials useful during a visit to specific places as well as information about cultural events [124–130]. In this way, tourists can easily access the cultural content, choosing the communication format they prefer supported by the technology they have. Another group of applications is implemented for educational purposes to support both teaching and rescuing the socio-cultural heritage [131–133]. The studies also include virtual libraries and platforms supporting 3D reconstruction. Another type of applications concerns the interactive multimedia frameworks for digital heritage narratives and storytelling as well as designing the user interface and the appearance of 3D model showing the described intangible heritage [134]. Applications dedicated to specific actions such as virtual archaeology or ontology are also created [135, 136].

To meet the requirements of software studies, the following technologies are usually used: 3D modelling, AR, VR, 3D visualisation and 3D scanning (Table 13). They allow to create intangible intellectual foundation, creative abilities, cultural identity and history, which are based on digital expressions of culture and identity.

**Table 13:** Technologies used in software system studies for mixed culture heritage

Papers	Technologies used
Pietroni et al. [124]	3D modelling, AR
Arias-Espinoza et al. [125]	3D modelling, AR, 3D visualization
Medina-Carrion et al. [126]	3D modelling, AR, 3D visualization
Viinikkala et al. [127]	AR, VR
Wen et al. [128]	AR, 3D modelling
Medici et al. [131]	3D scanning
Munster et al. [132]	3D modelling
Pettoello et al. [135]	VR, 3D visualization
Wu et al. [133]	3D modelling
Adabala et al. [134]	AR
Damiano et al. [136]	3D modelling, 3D visualization
Banfi et al. [129]	3D modelling, AR, VR
Ermrnyi et al. [130]	3D scanning, 3D modelling
Medici et al. [131]	3D scanning
Munster et al. [132]	3D modelling
Wu et al. [133]	VR
Adabala et al. [134]	AR
Pettoello et al. [135]	VR
Damiano et al. [136]	3D modelling, 3D visualization, VR

## Results

### Quantitative analysis

**Fig. 6:**
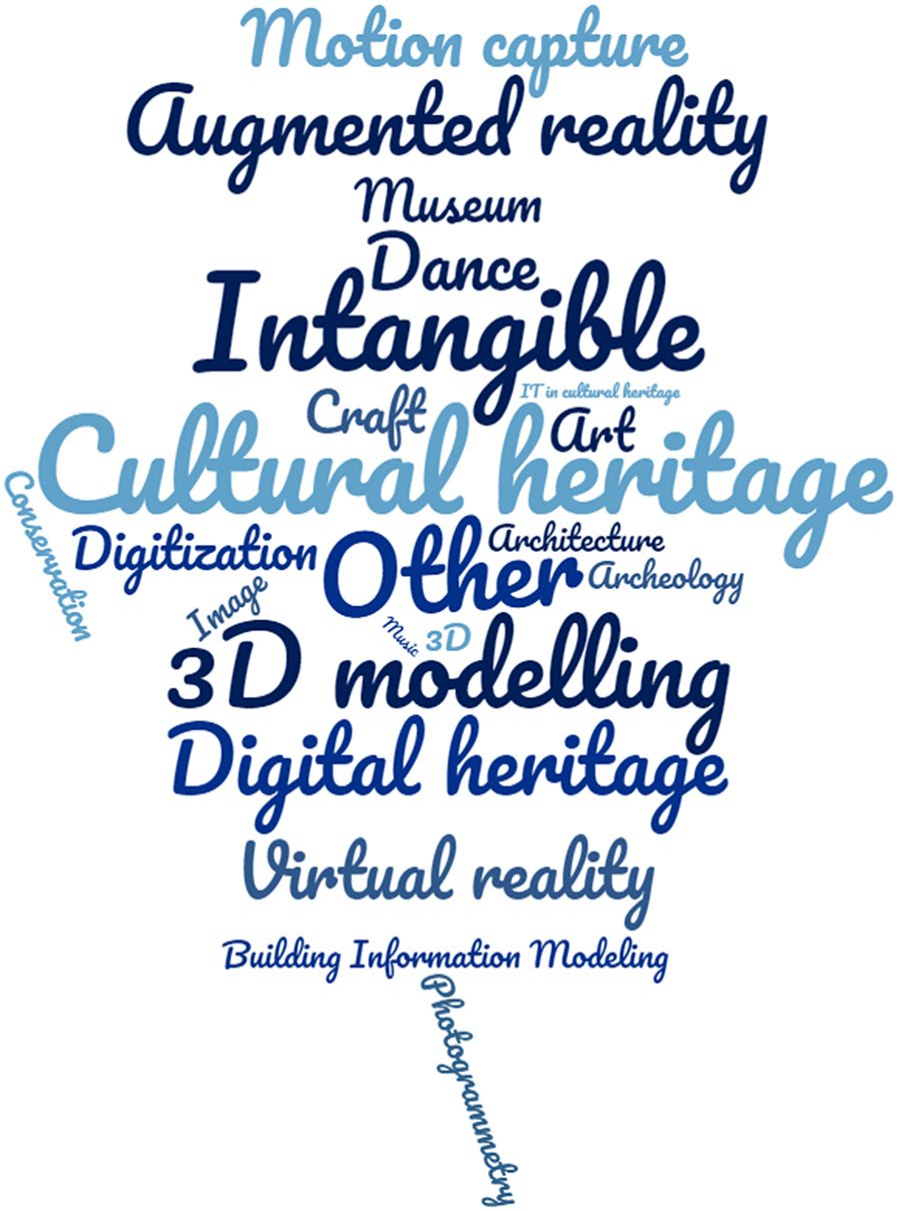
The cloud of given keywords in ICH papers

See Tables 14, 15, and Fig. 6.

**Table 14:** Analysed papers in the following databases in years 2005-2021

	Searched papers	Excluded papers	Included papers
Scopus	151	40	111
Web of Knowledge	113	23	90
IEEE Xplore	41	15	26

**Table 15:** Papers of tangible and intangible types for three databases

	Scopus	Web of knowledge	IEEE xplore
TCH	12	19	4
ICH	64	46	14
Mixed CH	35	25	8

### Detailed results

See Figs. 7, 8, 9, 10, 11, 12, 13, 14, 15 and 16.

**Fig. 7:**
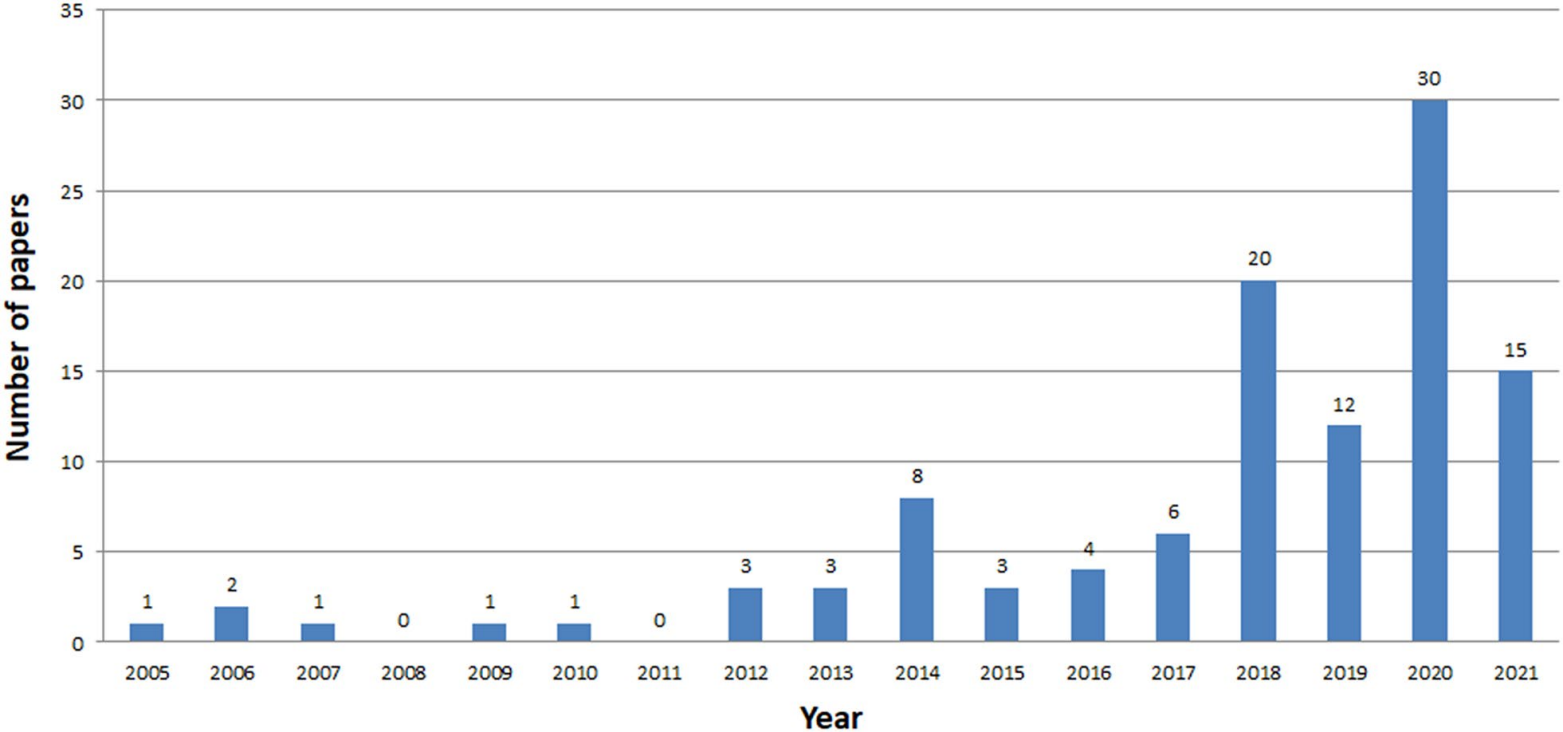
Number of ICH papers in Scopus in years 2005-2021

**Fig. 8:**
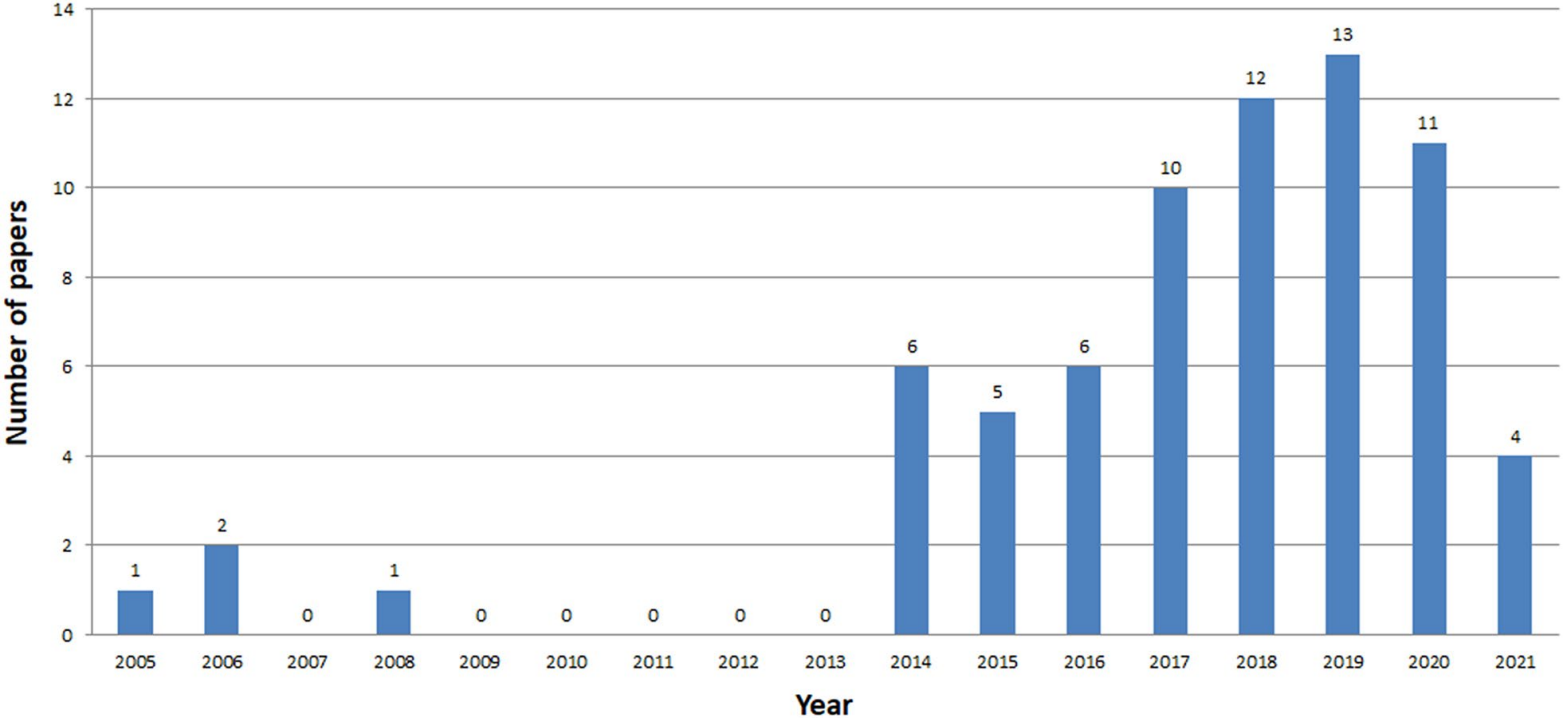
Number of ICH papers in Web of Knowledge in years 2005-2021

**Fig. 9:**
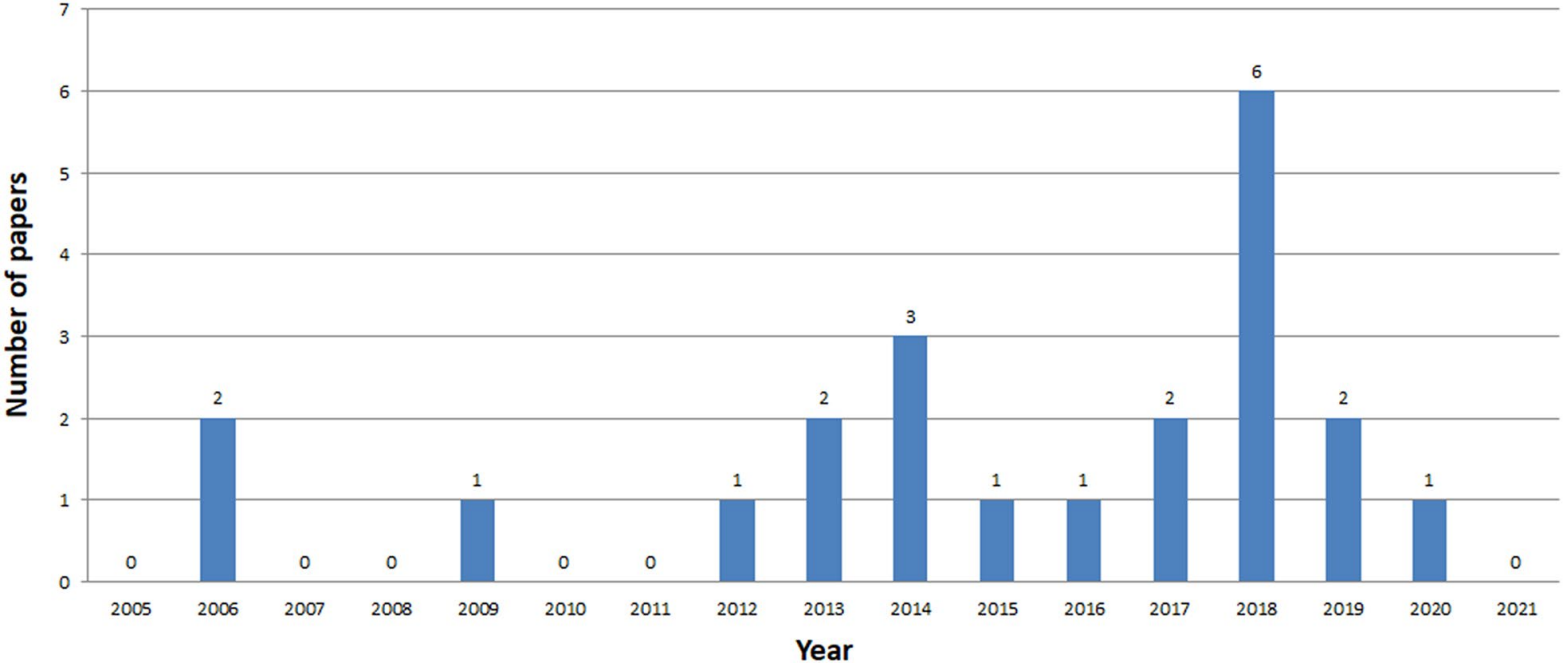
Number of ICH papers in IEEE Xplore in years 2005-2021

**Fig. 10:**
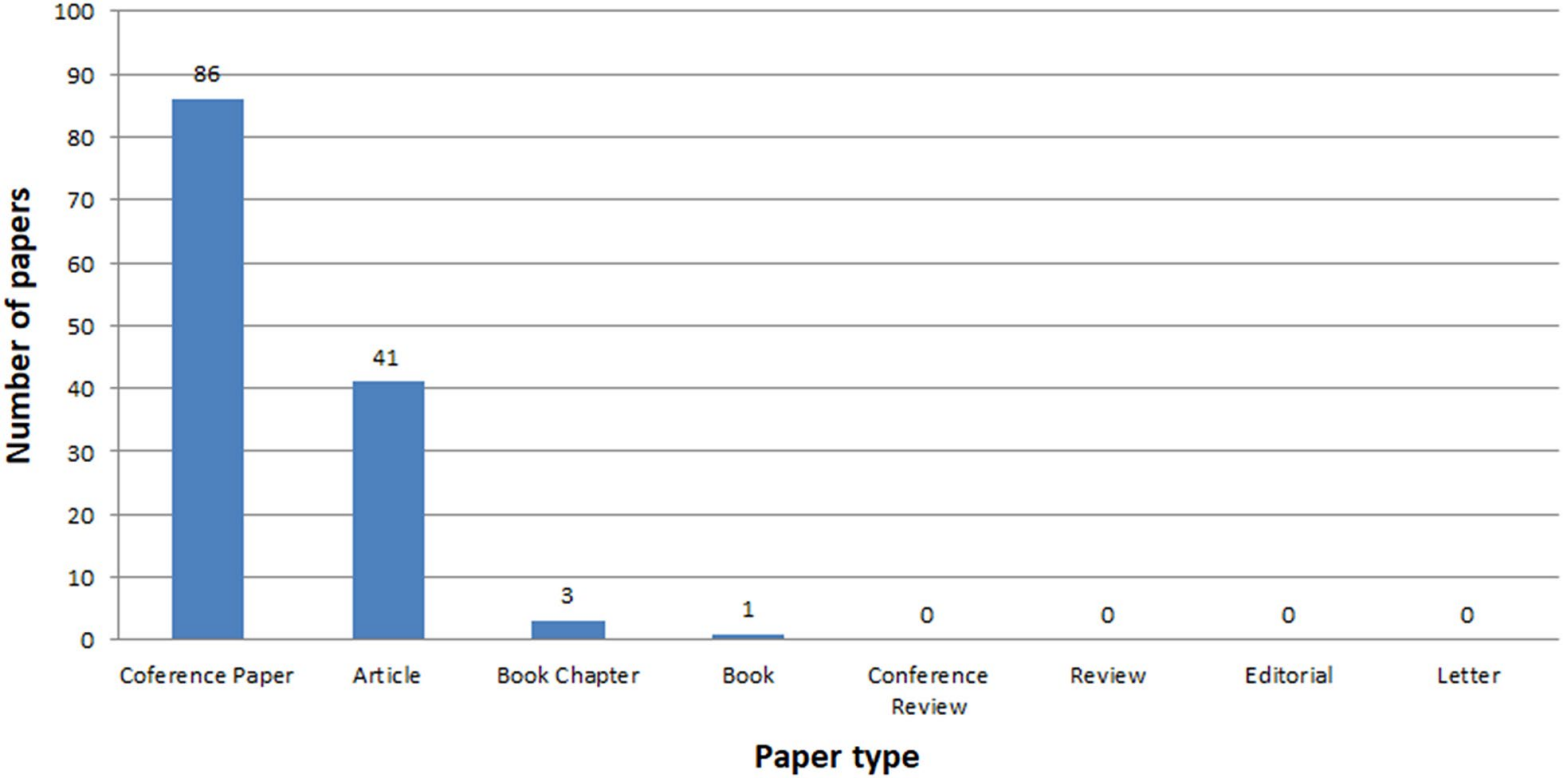
Number of ICH papers for selected types in years 2005-2021

**Fig. 11:**
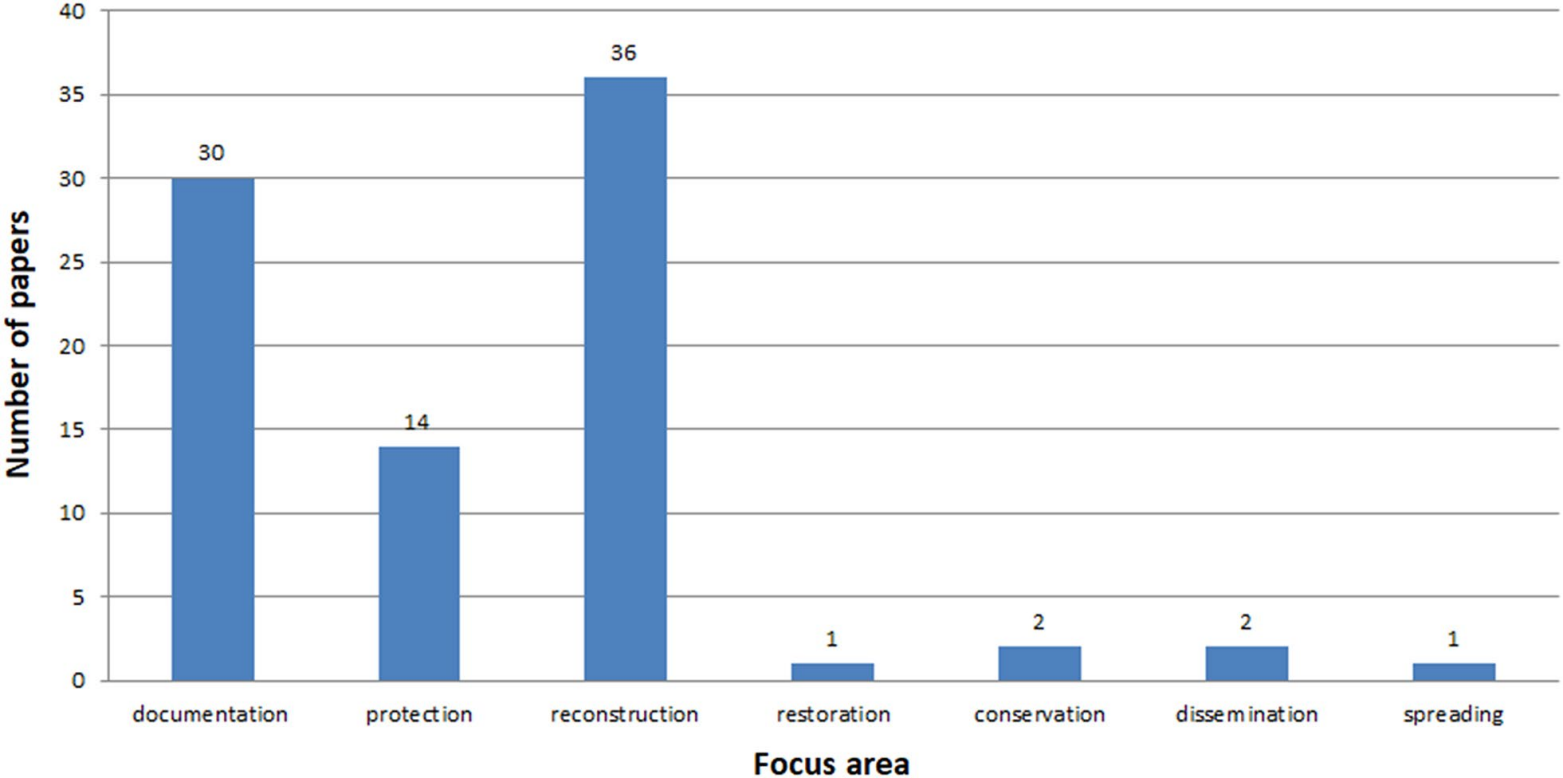
Focus areas presented in conference papers

**Fig. 12:**
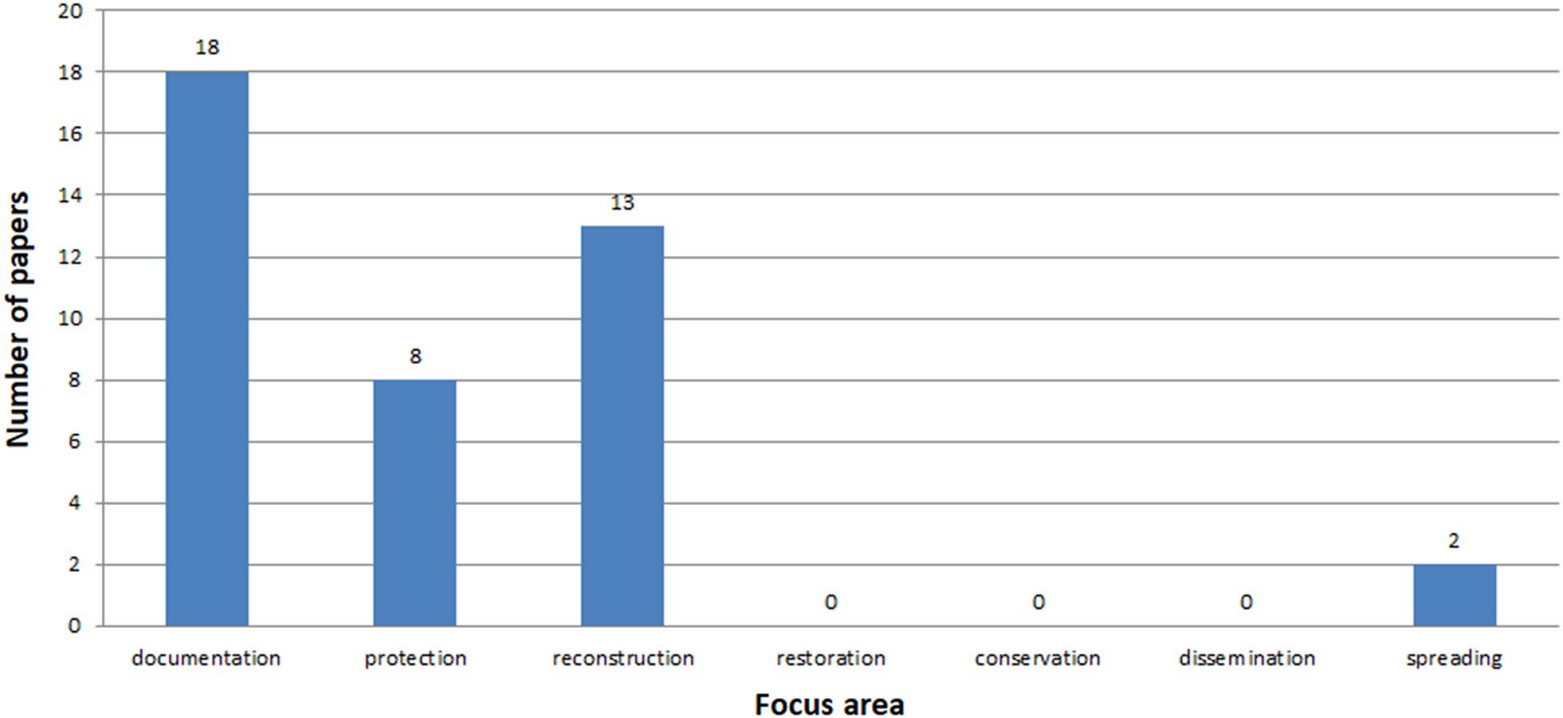
Focus areas presented in articles

**Fig. 13:**
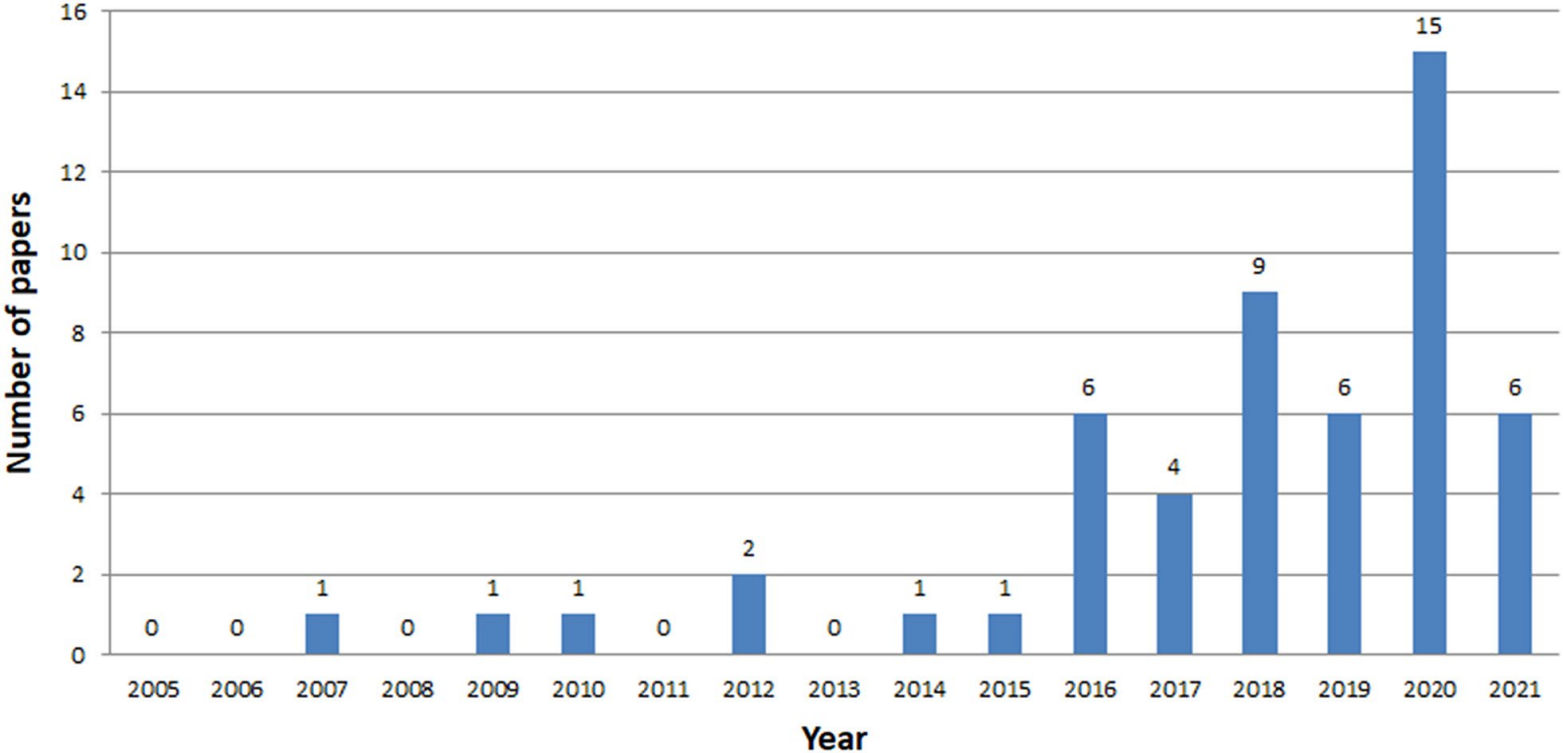
Number of studies concerning documentation focus area

**Fig. 14:**
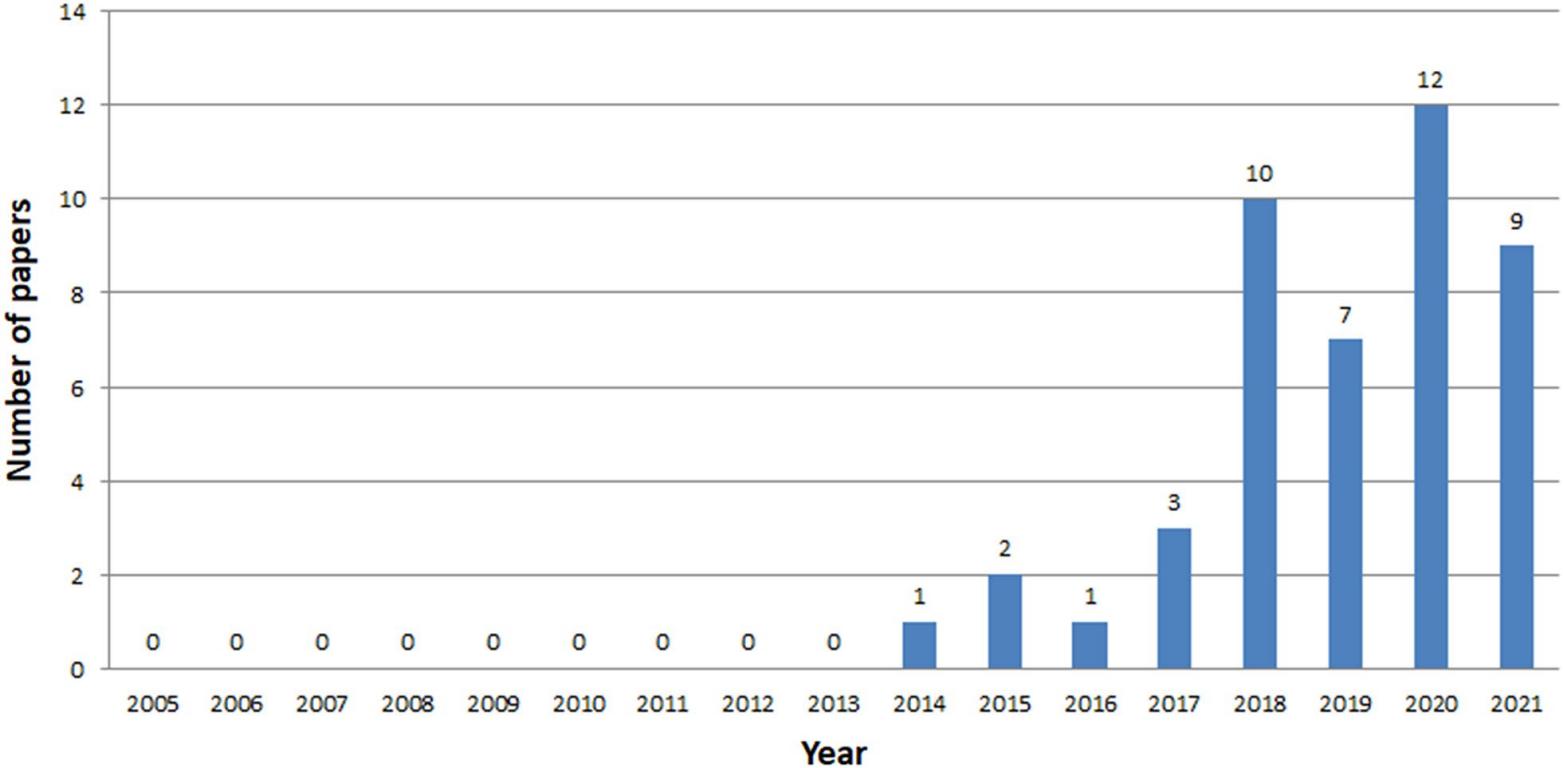
Number of studies concerning reconstruction focus area

**Fig. 15:**
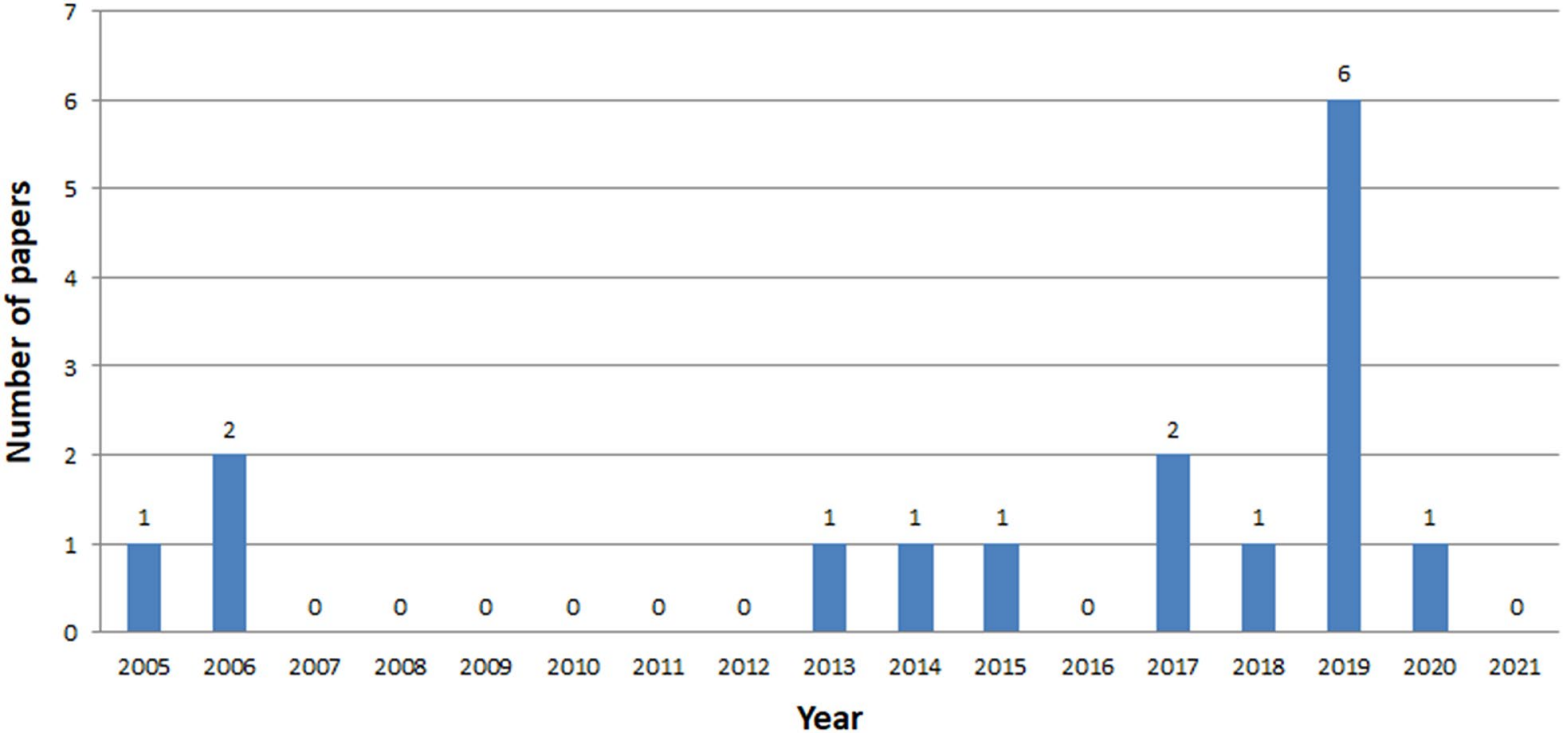
Number of studies concerning protection focus area

**Fig. 16:**
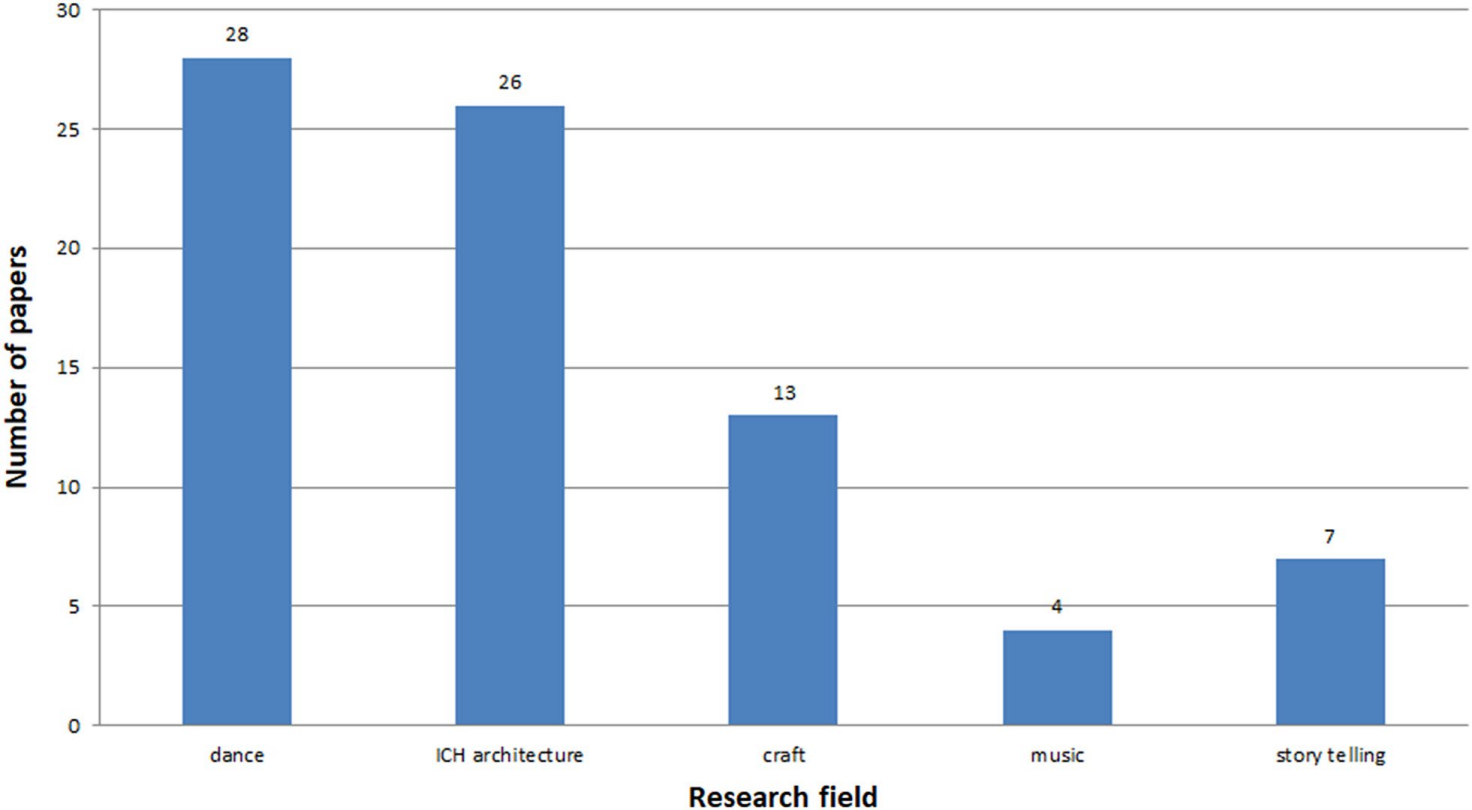
Number of studies concerning the following fields

### Geographic, technological and product cross-section

**Fig. 17:**
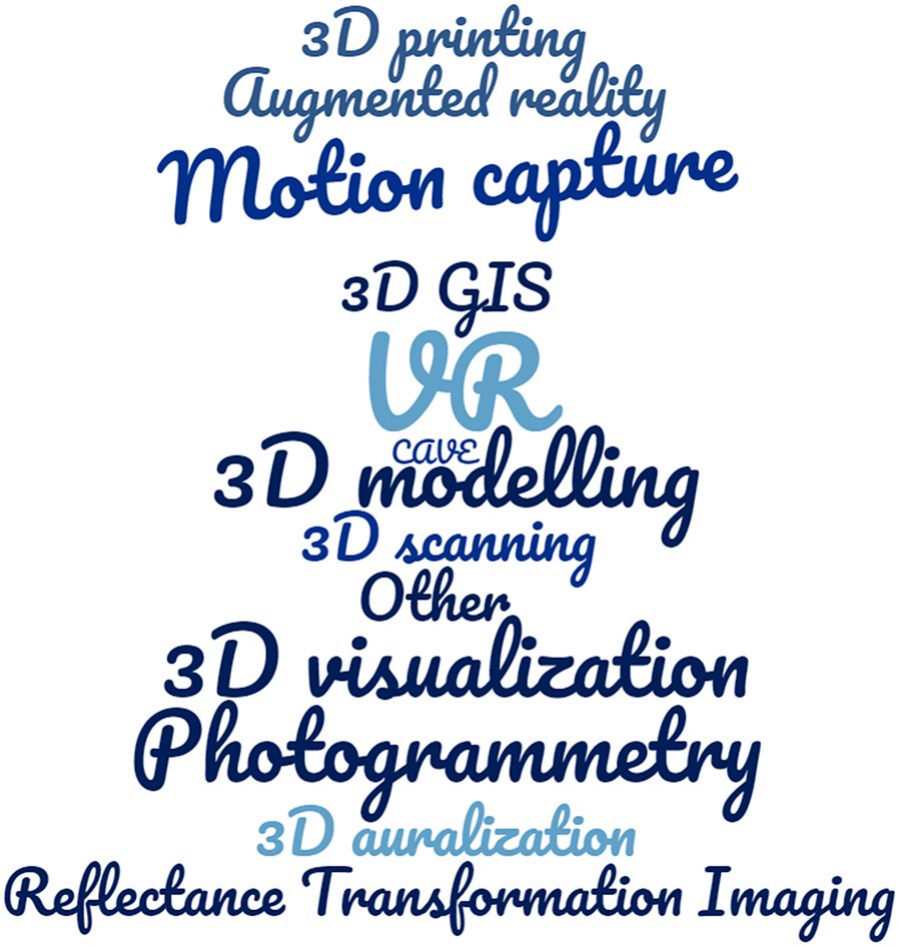
The cloud of 3D technologies used in the papers concerning intangible heritage

**Fig. 18:**
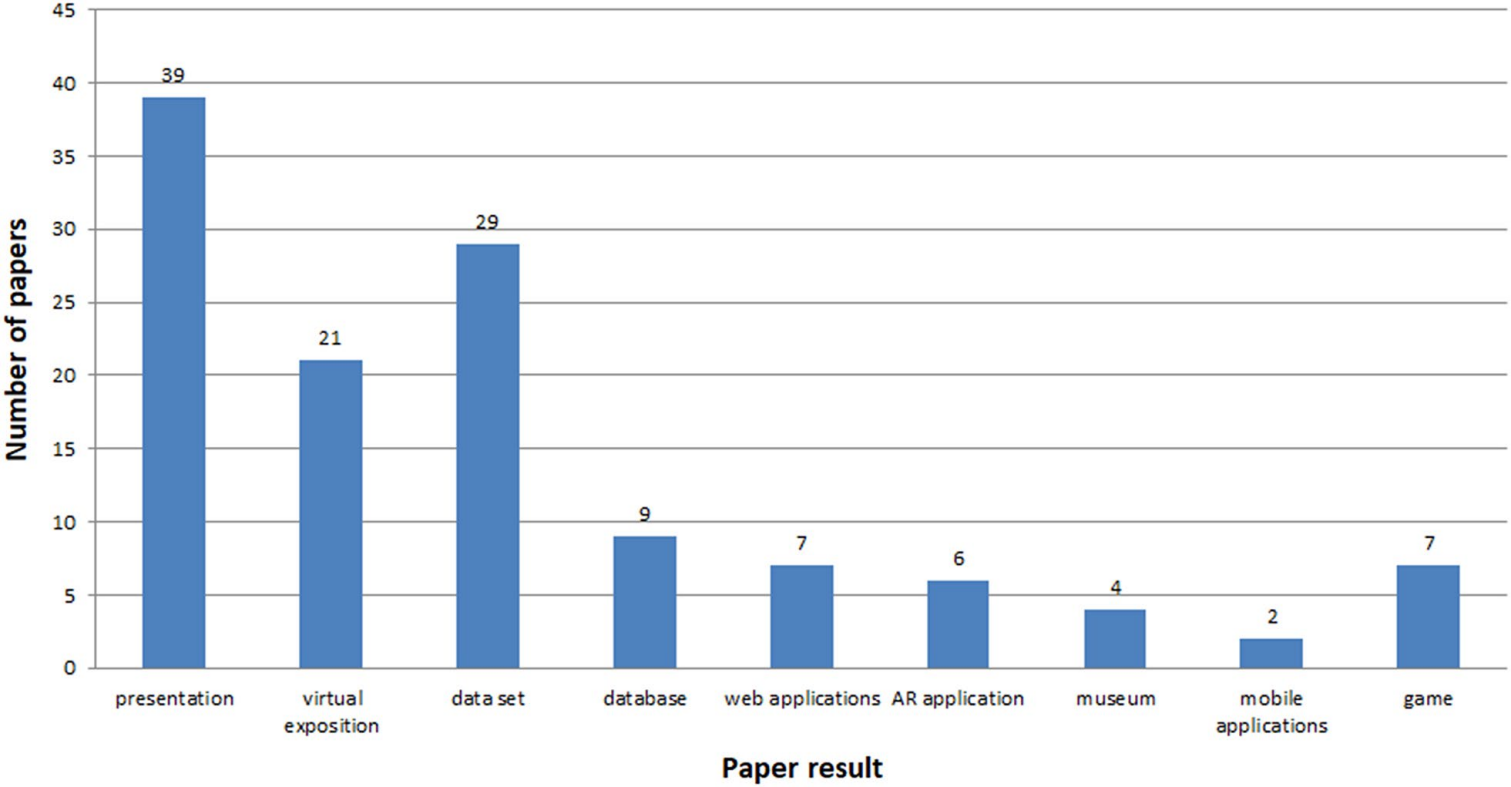
Number of papers concerning various results

See Tables 16 and 17, Figs. 17 and 18.

**Table 16:** Studies referring to countries

Country	Number of countries affiliation	Number of countries referred to in studies
China	36	31
Greece	29	23
Italy	22	13
Bosnia and Herzegovina	7	4
USA	6	1
Spain	4	3
India	4	3
Thailand	4	4
Turkey	4	0
Japan	5	4
France	2	2
The Netherlands	2	1
Ecuador	2	0
Korea	3	3
Hong Kong	1	0
Canada	2	0
Czech Republic	1	0
Slovenia	1	0
Lithuania	1	0
Portugal	1	0
Finland	1	1
Belgium	1	0
Switzerland	1	0
Germany	1	0
Ireland	1	1
Cyprus	1	1
Romania	0	1
Canari	0	2

**Table 17:** Number of studies for the specified aims

Aim of the study	Number of papers	Percentage [%]
Education	41	17.67
3D models	36	15.52
Promoting heritage	33	14.22
Learning	20	8.62
Virtual Reality	15	6.47
Recognition	17	7.33
Visual reconstruction	18	7.76
Identify the differences	11	4.74
Entertainment	15	6.47
Assessment	8	3.45
Create artwork	8	3.45
Analysis	4	1.72
Virtual tours	5	2.16
Storytelling	1	0.43

## Discussion

In this paper a multi-aspect analysis of 3D technologies in the aspect of both ICH and mixed CH is performed. In Table 14 the number of searched studies for the following TITLE-ABS-KEY text: *cultural AND (intangible OR non-material) AND heritage AND (3D OR three-dimensional OR “three dimensional”)* is shown. The papers to which there was no access in their entirety or which did not cover the specified topics of 3D technology or culture heritage were not taken into further consideration. Totally, 132 papers were considered for the analysis (Table 15). The first database, Scopus, contains the greatest amount of papers in this area (111). All papers searched in IEEE Xplore coincided with those in the Scopus database. 21 papers were found in the Web of Knowledge that were not in other databases.

The most popular 3D technologies used in ICH are (respectively): 3D modelling, motion capture, 3D visualisation, VR and AR. They constitute almost 86% of all technologies in the analysed publications (Tables 2, 3, 4, 5, 6, 7, 8 and 9). On the other hand, in mixed CH, the most commonly used 3D technologies turned out to be (respectively): 3D modelling, 3D visualization, VR and AR. They constitute almost 68% of all technologies in the analyzed publications (Tables 10, 11, 12 and 13).

3D model generation of artifacts, monuments or large environments is nowadays often used in scientific studies. 3D modelling is based on photographs and geometric projections according to architectural criteria [138]. It allows to create a digital representation of an object in three dimensions using dedicated software (e.g. Unity 3D, Maya, Auto CAD, 3D Studio Max). The most important advantages of 3D modelling include: easy to use tools with mathematical models, which does not require too much training, the possibility to work with complex items convenient for many types of objects and used in VR and AR as well as open-source software availability. Among the disadvantages of 3D modelling are the facts that it is not always possible to obtain fully natural shapes, it requires large computational resources and processing of complex models needs the use of commercial software. Nowadays 3D models must reach a sufficient level of realism and accuracy [137]. 3D modelling is applied in many fields: e. g. entertainment, gaming, ICH and CH.

**Fig. 19:**
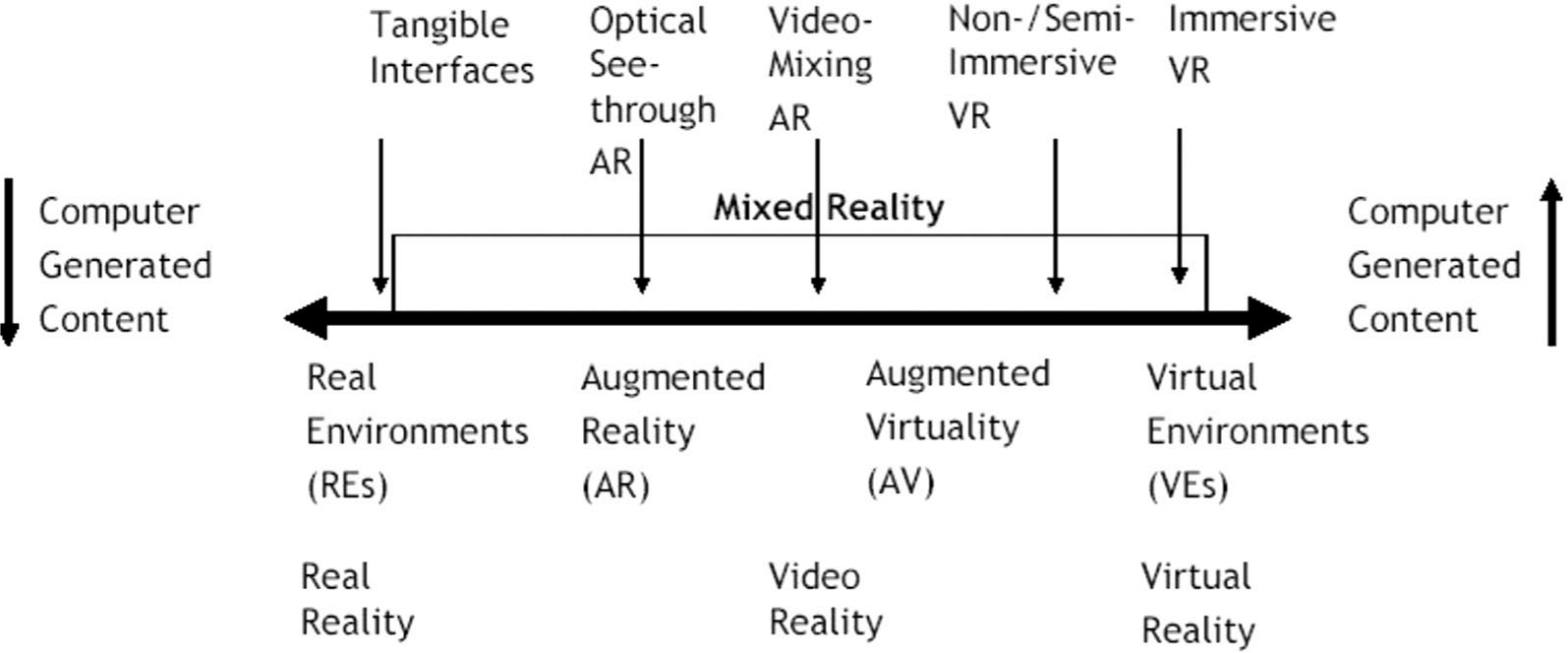
Reality-virtuality Continuum [140]

3D visualization is closely related to modelling. It makes possible for the analysts to gain a deeper, more intuitive understanding of the models. The development of Information Technology (IT) tools make it possible to present 3D models. Nowadays, non destructive techniques for creating 2D and 3D digital data become more and more useful for investigating ICH [138]. The visualization system should be able to display the field measurements and verification of 3D physical units. WebGL, GL Scene, OpenGL, Cesium JS, Google Earth, Computer-aided design (CAD), BIM or 3D map are commonly used visualisation platforms. 3D object management is possible through a dedicated user interface [139]. 3D visualization greatest advantages include: ease of implementation on platforms with raster graphics, precise and accurate rendering, ease of its distribution. The disadvantages of 3D visualization include: problems with implementing perspective and texture, expensive processing, difficulty in defining image depth. Reality-virtuality relationship, which integrates the most commonly used technologies in ICH, from the creation of 2D and 3D models, thought their visualisation as well as the creation of AR and VR applications, is presented in Fig. 19. AR applications are located closer to the real world, while the virtual world is represented by VR solutions.

AR is a technology that combines the interactive real world with an interactive computer-generated world in such a way that they together appear as one environment [140, 141]. It means that the user moves around the real object, while the virtual ones react in a way as it is integrated with the real world. The virtual object may move but they should reflect real-world movement rules.

The classification of AR systems may refer to many criteria, such as: hardware (e.g. the type of tracking system), visualisation approaches (see-through, video-mixture) or working distance (indoor, outdoor), communication (wireless, hardwired) [140]. Indoors systems use static markers that are attached to visible parts. They are limited to small 3D models, number of images or size of the user path. Outdoors system are based on markers that exist in the real world. The weather and its changes (e.g. sun shinning or shadows) is one of the biggest problems in this type of applications. Indoor systems provide accuracy of a few centimetres while outdoor ones need to use absolute or relative positioning systems, in combination with vision systems when the accuracy is not sufficient. Outdoor applications usually need a special transmission channel, which either has to be developed or existing communication services have to be used [140, 141].

VR is a modern technology that enables transfer to a virtual world that allows users to represent various elements, including culture. It may be defined as a way for humans to visualise, manipulate and interact with computers and complex data [140]. It consists of two important issues: the world (usually 3D) and an appropriate level of interaction with realism. VR systems may be classified based on [140]: hardware (from desktop equipment to spatially immersive systems, e.g. CAVE) and display system (from observing the screen, to being inside the VR environment) or both. Another type of classification is the type of simulation: pure geometry (lines, points, geometric shapes), static semantics (realistic complex static objects) and dynamic semantics (dynamic objects). These systems may be also divided based on the way data is displayed, such as: single frames, sequence of frames (animation), or real-time work. Interaction, which ranges from none to full immersion, indicates the special high level hardware equipment used (e.g. CAD or GIS). VR systems generally track the motion of hand-held objects or a user’s head or limbs, and the received data is used to determine the user’s view, navigation, interaction with objects, and possible movement of a virtual body [142]. One of the problem that VR needs to update the 3D imagery in real-time presents obvious challenges that have limited the ability to reproduce complex phenomena such as light refraction [142]. Another issue is eliminating latency, which requires to respond to the user’s movements in milliseconds.

Motion capture is a widely-used technique for capturing movements for digital processing. It can be divided for mechanical, magnetic and optical ones [143]. Mechanical systems use an exoskeleton consisting of encoders that record the rotation of individual joints. Based on the calculated values and positions of the encoders, computers determine the movement of a person. These systems are not limited by space or the number of cameras. However, the exoskeleton limits the movements of the reordered persons. The accuracy of movement capturing depends on the encoders position and modelling of the skeleton. Magnetic motion capture is done through a field of electro-Magenta in which sensors are electrics. The optical motion capture systems consist of synchronized cameras that register the movement. They can be divided on these with and without markers (markerless). They register movement in 3D coordinate system. The cameras send signals which are reflected from the markers and which returned to the sources. Visibility of markers by at least two cameras ensures indicating a position of the marker in space. The minimum frequency of the recorded motion is 45 Hz. However, for the more dynamic movements the higher frequency should be set, at least 100 Hz. The big disadvantage of these systems is that the markers may disappear from the video while they are covered during movement. That is why, the positions of these markers are then interpolated during post-processing which is usually time-consuming. An example of a markerless system is the Kinect sensor, which records movement in 2D coordinate system [144]. The slower Kinect sensor has a sampling frequency of 30–37 Hz, and insufficient smoothing algorithms may have contributed to inability to properly capture the flexion and extension peak amplitudes. They are less accurate in relation to the optical systems based on markers, but they are also much cheaper and commonly used in scientific research.

It has to be assumed that all above presented technologies may be integrated for ICH purposes (Tables 2, 3, 4, 5, 6, 7, 8, 9, 10, 11, 12 and 13).

The first aspect of presented analysis covers the specified keywords in ICH papers (Fig. 6). **Cultural heritage**, specified as a keyword, was returned in the greatest number of studies. It is the most general term that corresponds to both TCH and ICH areas. The next keyword with a high frequency of occurrence is **intangible**, which limits the discussed type of cultural heritage. Used in studies, 3D technologies are also often given as keywords. In the analysis carried out, the most common were: **3D modelling**, **AR** and **motion capture system**. They are up to date technologies that have found their application in this niche field. **Dance** was listed as one of the most common keywords. This means that it is one of the key elements of 3D ICH and has been recognised as an essential archiving knowledge for future generations.

The analysis clearly shows that the interest in ICH is growing year by year, starting from 2014 (Figs. 7, 8, 9). The exception is a slight decline in interest in 2019 in the Scopus database, and in 2020 in the Web of Knowledge. This may be due to the inability to conduct studies due to COVID-19. The process of review may also be time consuming. The least results are found in IEEE Explore. Since 2018 only a few papers have been stored. Papers concerning ICH have started appearing in small numbers since 2005. The decline in interest in publishing in 2007-2013 is clearly visible. The greatest amount of papers were published between 2018 and 2020.

The vast majority of all papers are published as conference papers and journal articles (Fig. 10), whereas the articles make up half of the conference papers. ICH is a developing field of science where a great amount of the studies is published at various conferences. One reason for this is that the initial results are sent for conferences. The more sophisticated ones are published in journals. Analysis of conference papers shows that the authors largely present topics related to three areas: documentation, reconstruction and protection (Figs. 11, 12). Papers concerning issues related to restoration, conservation, dissemination and spreading are in a minority. A similar tendency is maintained in the works appearing in thematic journals. Here, also, the greatest emphasis is put on documenting ICH (Fig. 12). However, there were a few issues related to restoration, conservation, dissemination, and the number of works deals with spreading is 2.

The documentation area consists of various aspects of ICH: music [71, 96], tuna fishing [19], crafting [20, 21] storytelling [57, 60, 121], visualisation of historical sites in Iran [114], Art Gallery of Shanghai Style Lacquerware [55], capturing dance [33, 34, 40, 41, 111], art [110], virtual exhibitions [62, 99, 100, 105, 145], 3D models [94, 146] and settlement [147, 148]. Oral interpretations and their expressions are also one of ICH type. They are used in game-based learning in virtual museums to document culture and encourage visitors to expand their knowledge in this area [62].

The reconstruction of various ICH items include: museums [3], traditional folk dances [35, 36, 49, 50, 52, 111], traditional crafts which contains a lot of valuable information on how ancestors made objects, which are forgotten by successive generations is presented in [23, 72]. 3D technology allows to present ancient festivals and is unique in ICH: Lantern Festival [58], “Noh” and “Kabuki” [149]. The virtual reconstruction of the ancient internal flame lighting systems is presented in [93]. The research was used for defining the lighting scenarios according to various historical-interpretative hypotheses and for reconstructing of the luminaires. The reconstruction of ancient buildings are described in [102, 103, 122, 125, 126, 150], ancient routes in [116] and ancient art of war in [151].

In [95] the application and research in ICH Quanzhou marionette protection is described. Based on the folk intangible cultural heritage centre the interactions were created to protect ICH [73]. Other studies which concern protection topic are presented in [3, 30, 112, 118, 119, 149, 152].

The most applied focus areas, documentary, protection and reconstruction were divided into the years of the publication (Figs. 13, 14 and 15). The studies concerning protection started in 2005, the documentary in 2007 and the reconstruction in 2014. The most papers were published in the years 2016-2020. It means that intangible heritage is a developing field of study.

Analysing the aspects described in the papers, it can be stated that there are two very important issues of the human culture: dance and ICH aspects of architecture (Fig. 16). It has been noticed that these aspects of ICH culture are highly important, up to 73% of all studies. It seems that these two fields of study have a great potential. Due to sophisticated modern technologies dance may be modelled and its intangible aspects passed down to future generations. It should be emphasized that these fields of study also appear in other types of papers concerning software, game-based learning and others. In the analysed studies the most important technologies are: VR, 3D modelling, 3D visualisation, motion capture and Photogrammetry (Fig. 17).

The greatest amount of studies is performed in China, Greece and Italy. These studies are done by the domestic and foreign researchers in multi-disciplinary groups. These are countries with a great potential of culture. The aims of the studies vary (Tables 16 and 17). However, the most common purpose was education (17.67%), creation of 3D models (15.52%) and promotion of heritage (14.22%). An important issue was the element of learning and VR creation (Figs. 18, 19).

Many models have been created using different tools and formats concerning cultural heritage. In 2013, an attempt was made to formalise the data for cultural heritage, which is presented in paper [153]. A generic, extendable and interoperable framework for the development of cultural heritage Spatial Data Infrastructures (SDIs) was proposed. It is designed to be an extension of the Protected Sites Data Specification, in order to enable a full integration of cultural heritage data. Due to the fast digital development there are many attempts to standardise cultural data (e. g. 3D digital models of sculptures, monuments, rooms, buildings and audiovisual data: music, film or stage performances). In Germany the idea of such a framework is developed by the NFDI4Culture consortium [154].

## Conclusions

In this paper a multi-aspect analysis of 3D technologies in the aspect of both ICH and mixed CH for culture preservation is performed, based on three databases: Scopus, Web of Knowledge and IEEE Xplore. The performed analysis consisted of three main areas: general quantitative studies, detailed results and geographic, technological and product cross-section. In the paper, four questions were defined/stated in the Study questions section. Based on the obtained results it can be confirmed that the use of 3D digital technologies is increasing year by year. The results showed that the most important ICH-focused topics presented in the research are: dance, ICH architecture and crafts. The interest in folklore dances and the need to consolidate them is clearly visible. They are used both for educational and scientific purposes. Currently, there is a growing interest in the method of producing everyday objects and the methods implemented in the construction of buildings in the past. The study carried out showed that the most popular 3D technologies used in ICH are: 3D modelling, motion capture, 3D visualization, VR and AR. The current rapid development of this technology allows scientists to permanently consolidate such a valuable and elusive culture that is passed on to the next generations, often fading away or being distorted. Most of the articles concern ICH in the areas of data documentation, data protection and reconstruction. They concern articles published in scientific journals and conference materials. There is a noticeable increase in publications from year to year. The preservation of cultural heritage has become an anchor of the past towards future generations due to 3D technologies.

The obtained results showed that ICH is an indispensable extension of TCH. It is visible in the growing interest of ICH in the scientific studies. The society is aware of ICH loss, which is seen in the increasing number of research papers.

3D technologies are used in a wide range of applications. They concern not only the preservation of objects from the past, but also the development of today’s culture. Due to the development of 3D technology and IT, it can be expected that they will be increasingly used in ICH issues. Their usage in various types of applications will allow the spread of knowledge about cultural heritage. This will enable for the preservation of many elements of culture, both tangible and intangible, that will be available to present and future generations.

## Data Availability

Not applicable.

## Abbreviations

CH: Cultural heritage
TCH: Tangible cultural heritage
ICH: Intangible cultural heritage
3D: three-dimensional
VR: Virtual reality
AR: Augmented reality
